# ‘Trapped re-entry’ as source of acute focal atrial arrhythmias

**DOI:** 10.1093/cvr/cvad179

**Published:** 2023-12-04

**Authors:** Tim De Coster, Alexander S Teplenin, Iolanda Feola, Cindy I Bart, Arti A Ramkisoensing, Bram L den Ouden, Dirk L Ypey, Serge A Trines, Alexander V Panfilov, Katja Zeppenfeld, Antoine A F de Vries, Daniël A Pijnappels

**Affiliations:** Laboratory of Experimental Cardiology, Department of Cardiology, Leiden University Medical Center, Albinusdreef 2, PO 9600, 2333 ZA Leiden, The Netherlands; Laboratory of Experimental Cardiology, Department of Cardiology, Leiden University Medical Center, Albinusdreef 2, PO 9600, 2333 ZA Leiden, The Netherlands; Laboratory of Experimental Cardiology, Department of Cardiology, Leiden University Medical Center, Albinusdreef 2, PO 9600, 2333 ZA Leiden, The Netherlands; Laboratory of Experimental Cardiology, Department of Cardiology, Leiden University Medical Center, Albinusdreef 2, PO 9600, 2333 ZA Leiden, The Netherlands; Laboratory of Experimental Cardiology, Department of Cardiology, Leiden University Medical Center, Albinusdreef 2, PO 9600, 2333 ZA Leiden, The Netherlands; Laboratory of Experimental Cardiology, Department of Cardiology, Leiden University Medical Center, Albinusdreef 2, PO 9600, 2333 ZA Leiden, The Netherlands; Laboratory of Experimental Cardiology, Department of Cardiology, Leiden University Medical Center, Albinusdreef 2, PO 9600, 2333 ZA Leiden, The Netherlands; Laboratory of Experimental Cardiology, Department of Cardiology, Leiden University Medical Center, Albinusdreef 2, PO 9600, 2333 ZA Leiden, The Netherlands; Laboratory of Experimental Cardiology, Department of Cardiology, Leiden University Medical Center, Albinusdreef 2, PO 9600, 2333 ZA Leiden, The Netherlands; Department of Physics and Astronomy, Ghent University, 9000 Ghent, Belgium; Biomed Laboratory, Ural Federal University, 620002 Ekaterinburg, Russia; World-Class Research Center ‘Digital Biodesign and Personalized Healthcare’, I. M. Sechenov First Moscow State Medical University, 119146 Moscow, Russia; Laboratory of Experimental Cardiology, Department of Cardiology, Leiden University Medical Center, Albinusdreef 2, PO 9600, 2333 ZA Leiden, The Netherlands; Laboratory of Experimental Cardiology, Department of Cardiology, Leiden University Medical Center, Albinusdreef 2, PO 9600, 2333 ZA Leiden, The Netherlands; Laboratory of Experimental Cardiology, Department of Cardiology, Leiden University Medical Center, Albinusdreef 2, PO 9600, 2333 ZA Leiden, The Netherlands

**Keywords:** Optogenetics, Computer modelling, Atrial arrhythmias, Fibrosis, Cell culture

## Abstract

**Aims:**

Diseased atria are characterized by functional and structural heterogeneities, adding to abnormal impulse generation and propagation. These heterogeneities are thought to lie at the origin of fractionated electrograms recorded during sinus rhythm (SR) in atrial fibrillation (AF) patients and are assumed to be involved in the onset and perpetuation (e.g. by re-entry) of this disorder. The underlying mechanisms, however, remain incompletely understood. Here, we tested whether regions of dense fibrosis could create an electrically isolated conduction pathway (EICP) in which re-entry could be established via ectopy and local block to become ‘trapped’. We also investigated whether this could generate local fractionated electrograms and whether the re-entrant wave could ‘escape’ and cause a global tachyarrhythmia due to dynamic changes at a connecting isthmus.

**Methods and results:**

To precisely control and explore the geometrical properties of EICPs, we used light-gated depolarizing ion channels and patterned illumination for creating specific non-conducting regions *in silico* and *in vitro*. Insight from these studies was used for complementary investigations in virtual human atria with localized fibrosis. We demonstrated that a re-entrant tachyarrhythmia can exist locally within an EICP with SR prevailing in the surrounding tissue and identified conditions under which re-entry could escape from the EICP, thereby converting a local latent arrhythmic source into an active driver with global impact on the heart. In a realistic three-dimensional model of human atria, unipolar epicardial pseudo-electrograms showed fractionation at the site of ‘trapped re-entry’ in coexistence with regular SR electrograms elsewhere in the atria. Upon escape of the re-entrant wave, acute arrhythmia onset was observed.

**Conclusions:**

Trapped re-entry as a latent source of arrhythmogenesis can explain the sudden onset of focal arrhythmias, which are able to transgress into AF. Our study might help to improve the effectiveness of ablation of aberrant cardiac electrical signals in clinical practice.

## Graphical Abstract

**Graphical Abstract:**
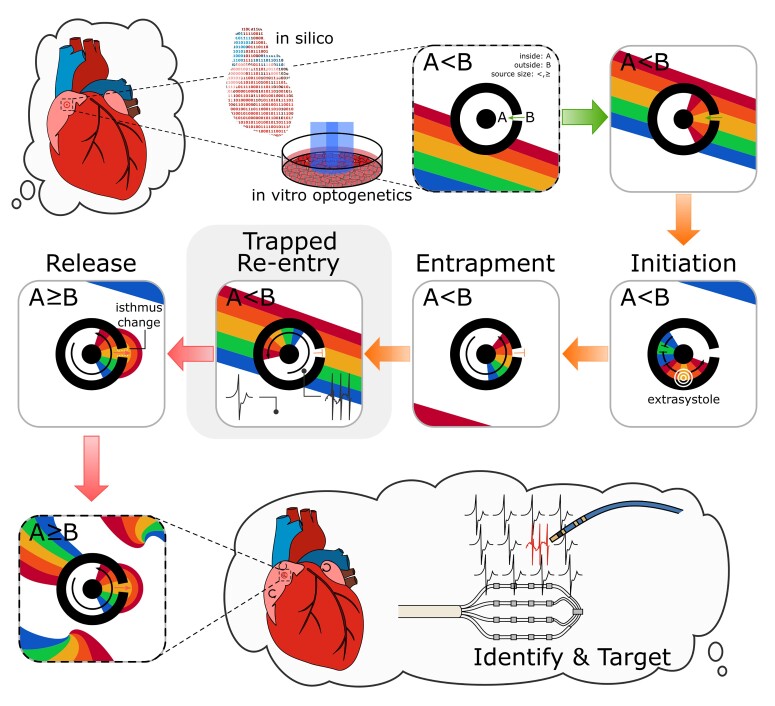

**Time of primary review: 35 days**

**See the editorial comment for this article ‘Latent drivers for atrial fibrillation and specific patterns of localized fibrosis’, by A.J. Rogers and S.M. Narayan, https://doi.org/10.1093/cvr/cvae032.**

## 1. Introduction

Quivering of the heart’s upper chambers during atrial fibrillation (AF) is associated with increased cardiovascular morbidity, mortality, and impaired quality of life.^1^ To improve AF-related therapeutic strategies, it is important to enlarge insight in the electrophysiological mechanisms underlying this cardiac arrhythmia.

Diseased atria are characterized by structural heterogeneities (e.g. fibrotic regions) and functional heterogeneities (e.g. areas of altered conduction not associated with an anatomical obstacle). Such areas of altered texture and conduction are revealed by abnormal electrograms or complex fractionated atrial electrograms (CFAEs).^2^ The regions in which these CFAEs occur often display an increased collagen content [(micro)fibrosis] and myocardial fibre dissociation.^3^ The spatial distribution of these structural changes rather than the amount of collagen is the major determinant of the occurrence and appearance of CFAEs.^4^ Pathological distributions of structural or functional heterogeneities produce differences in electrical load in the conduction pathway, creating ‘non-uniform anisotropic’ impulse propagation.^5^ They add to abnormal impulse generation and propagation, originating from e.g. ectopy^6^ and unidirectional conduction block,^7^ creating various sources of re-entrant activity, which lead to arrhythmias including AF. These arrhythmogenic conduction events can occur in very small areas (e.g. re-entrant circuits as small as 0.6 × 2.6 mm) in human atrial bundles.^8,9^

AF can be sustained by localized sources in the form of electrical rotors and focal impulses, for which catheter ablation improves clinical outcome.^10–12^ These sources conceivably correlate with areas of atrial fibrosis in which the site-specific micro-architecture of connective tissue fibres and the remaining myocardial fibres allow re-entrant activity to occur and sustain.^8^ The elimination of CFAEs by single ablation lesions,^13^ which typically have a diameter of 5–7 mm, is in accordance with the possibility that very small re-entrant circuits underlie CFAEs.

CFAEs have been selected as targets for catheter ablation to treat AF,^10,14^ leading to a one-year success rate of up to 91%. Although it has been suggested that these electrograms may represent areas of slow conduction or pivoting points in circuits of re-entry associated with AF, the mechanism underlying the various types of CFAEs has not been fully elucidated.^15^ One of these cases of which the origin is not clear, was the detection of CFAEs during sinus rhythm (SR).^16^

Here, we provide an explanation for such CFAE observations that might lead to sudden arrhythmia initiation. This explanation merges micro-re-entry and source-sink mismatch. Using computer simulations and monolayers of optogenetically modified [i.e. Ca^2+^-translocating channelrhodopsin (CatCh)-expressing^17^] neonatal rat atrial myocytes (NRAMs)^18^ to create structural heterogeneities, we show that a source of re-entrant excitation can be electrically shielded from the remaining part of the atria. The electrical separation of the source of re-entrant excitation can occur due to current-to-load mismatch^19^ between the shielded region and the remaining atrial tissue. When this source is electrically shielded, it will coexist with normal heart beats and thus induce CFAEs during SR. However, some factors, e.g. partial cellular uncoupling^20^ can reduce that current-to-load mismatch and the initially isolated re-entrant wave will start to affect the rest of the atria causing an atrial arrhythmia. Due to the fact that the re-entrant source can lock itself away from the rest of the atrial tissue, we have coined it ‘trapped re-entry’.

## 2. Methods

Expanded methods can be found in the Supplementary material online, *Methods* (Sections 1.1–4.7) and *Major Resources Table*.

### 2.1 Experimental methods

All animal experiments were reviewed and approved by the Animal Experiments Committee of the Leiden University Medical Center (AVD116002017818 and AVD15460) and performed in accordance with the recommendations for animal experiments issued by the European Commission directive 2010\63.

#### 2.1.1 Preparation of neonatal rat atrial monolayers

Two-day-old Wistar rats (RRID:RGD_737929) were sedated through isoflurane (2–3%) inhalation, after which the animals were decapitated and their heads were submerged in liquid nitrogen to stop brain activity. Subsequently, the hearts were excised and atrial cells were isolated to establish NRAM monolayers.

#### 2.1.2 Optical voltage mapping of monolayers of CatCh-expressing NRAMs

NRAM monolayers were transduced with a lentiviral vector encoding the depolarizing light-gated ion channel CatCh resulting in transgene expression in nearly 100% of the cells.^21^ Only monolayers showing uniform action potential propagation upon 1 Hz electrical pacing were used (*n* = 13). Optical voltage mapping was performed with a MiCAM05-Ultima camera (SciMedia, Costa Mesa, CA), measuring a 100 × 100 pixel image covering an area of either 1.65 × 1.65 cm^2^ (*n* = 8) or 1.01 × 1.01 cm^2^ (*n* = 5) at a frame rate of 167 Hz. The resulting images were used to investigate entrapment and escape of excitation waves in the CatCh-expressing monolayers by using a patterned illuminator connected to a 470 nm LED source,^18^ resulting in a light intensity of 30 mW/cm^2^ at the upper cell surface.

#### 2.1.3 Optical pattern design

For the NRAM monolayers, the circuit of trapped re-entry was created between two separate non-conducting circular regions in wells of 24-well cell culture plates. The inner circle had a diameter of 0.26 cm; the inside and outside diameter of the surrounding ring were 0.65 and 0.93 cm, respectively. The area of optogenetically imposed conduction block shown in *Figure 2G* measured 1.467 × 0.187 cm^2^ (height × width), with centrally localized funnels varying in width from 0.244 to 0.041 cm.

#### 2.1.4 Stimulation protocol

A trapped wave was created inside the optogenetically isolated region of conduction by programmed optical stimulation (S1S2 protocol, Supplementary material online, *Figure S1*). During this process, periodic electrical stimulation was applied from the bottom left of the culture, mimicking SR in the atria. These pulses were delivered at a frequency of 1 Hz through an epoxy-coated bipolar platinum electrode delivering square 10 ms, 8 V suprathreshold electrical impulses via a STG 2004 stimulus generator and MC Stimulus II software (both from Multi Channel Systems, Reutlingen, Germany) and were continuously applied.

### 2.2 Computational methods

The transmembrane voltage (V) was calculated in millivolts (mV), evolving spatiotemporally and obeying the reaction-diffusion equation:


(Eq.1) $$\frac{\partial V}{\partial t}=\nabla (D\nabla V)-\frac{I_{\mathrm{ion}}+I_{\mathrm{stim}}}{C_{m}}$$

where *t* is time in milliseconds (ms), *I*_ion_ is the total ionic current density in microampere per square centimetre (μA/cm^2^), *I*_stim_ is the external stimulus current in μA/cm^2^, *C_m_* is the specific membrane capacitance in microfarad per square centimetre (μF/cm^2^), and **D** is the diffusion tensor, which determines the electrical conductivity of cardiac tissue in each direction of propagation, i.e. the mathematical representative of gap junctional coupling efficiency.

#### 2.2.1 Simulations of NRAM monolayers

The electrophysiological properties of NRAMs in a homogeneous monolayer were modelled according to Majumder *et al.* ^22^ The optogenetic tool used in the numerical studies was a previously described model of *Chlamydomonas reinhardtii* channelrhodopsin-2 mutant H134R.^23^ In order to demonstrate the trapping of a re-entrant wave, we designed several illumination patterns that were projected onto our *in silico* monolayers. From the centre to the periphery they consisted of an illuminated circle, a ring of unexposed tissue and a ring of illuminated tissue with strategically positioned funnels (isthmi) with varying opening angles.

#### 2.2.2 Simulations of human atria

Anatomical data and fibre directions of human atria were obtained from Dössel *et al.* ^24^ for realistic anisotropic simulations. The three ionic cell models that were used are the human atrial Courtemanche model,^25^ the Courtemanche model of AF-induced electrical remodelling,^26^ and the chronic AF-remodelled human atrial Loewe model^27^ (see Supplementary material online, *Figure S2*).

#### 2.2.3 Funnel opening design

Both in the two-dimensional (2D) and three-dimensional (3D) models, the design of the funnel-shaped connection between the circuit of trapped re-entry and the surrounding cardiac tissue was based on the underlying principles of source-sink mismatch.

#### 2.2.4 Circuit design

The circuits in the *in silico* and *in vitro* NRAM monolayers, had identical dimensions. The 3D *in silico* human atria were bounded by a box with dimensions of 10.755 × 8.895 × 7.035 cm^3^, which corresponds to the size of the atria of human adults.^28^ For the region of trapped re-entry, the size of the inner obstacle was 1.410 × 0.468 cm^2^ × local wall thickness and that of the circuit (i.e. inner obstacle plus conducting region) was 2.694 × 1.8375 cm^2^ × local wall thickness.

#### 2.2.5 Stimulation protocols

For *in silico* 2D monolayers of NRAMs, an S1S2 protocol was used to induce trapped re-entry. Once trapped re-entry was established, either no or 1 Hz bulk pacing was performed. All *in silico* 3D human atrial models were pre-paced 50 times at 1 Hz at the single cell level to start with stable cell parameters, after which they were used for whole atria simulations. To establish trapped re-entry, again an S1S2 protocol was used. For the S1 pulse, the sinus node was activated in the atria. The S2 pulse was timed inside the circuit of trapped re-entry. Once trapped re-entry was established, pacing continued from the sinus node at 1 Hz. Each simulation lasted 12 s.

#### 2.2.6 Electrogram computation

The extracellular potential *ϕ_e_* (for unipolar electrograms) was modelled using a current source approximation for a large volume conductor:


(Eq.2) $$\phi_{e}(x,t)=\frac{1}{4\pi \sigma_{e}}\int dy\frac{I_{m}(y,t)}{\left|x-y\right|}$$

where **x** is the electrode location vector, **y** is the current source location vector, *I_m_* is the transmembrane current per unit area of atrial tissue surface, and *σ_e_* = 5.34 mS/cm is the extracellular conductivity.^29^

### 2.3 Statistical analysis

Trapped re-entry was demonstrated in eight different monolayers by varying the timing of the S1 and S2 stimuli. Conduction velocities in these monolayers were compared using a paired Wilcoxon signed rank test. Conduction velocities were reported as average ± standard deviation, as indicated.

In digital simulations, release frequencies were measured once the escape rhythm had stabilized. Maximal errors on the digitally investigated parameter ranges mentioned in Supplementary material online, *Table T1* are the smallest step-size with which these parameters were changed.

## 3. Results

We demonstrate the principle of trapped re-entry by reproducing its three distinct and subsequent phases: (1) SR, (2) establishment of a trapped excitation wave not affecting SR in the surrounding tissue, and (3) transmission of re-entrant waves to the surrounding tissue. We show this principle in a simplified, fully controllable 2D *in silico* model of NRAMs, in an experimental *in vitro* model of optogenetically modified NRAM monolayers, and in a realistic 3D model, i.e. an *in silico* digital twin of whole human atria containing a fibrotic area capable of accommodating a re-entrant wave.

### 3.1 Trapped reentry in a 2D *in silico* model of NRAMs

**Figure 1:**
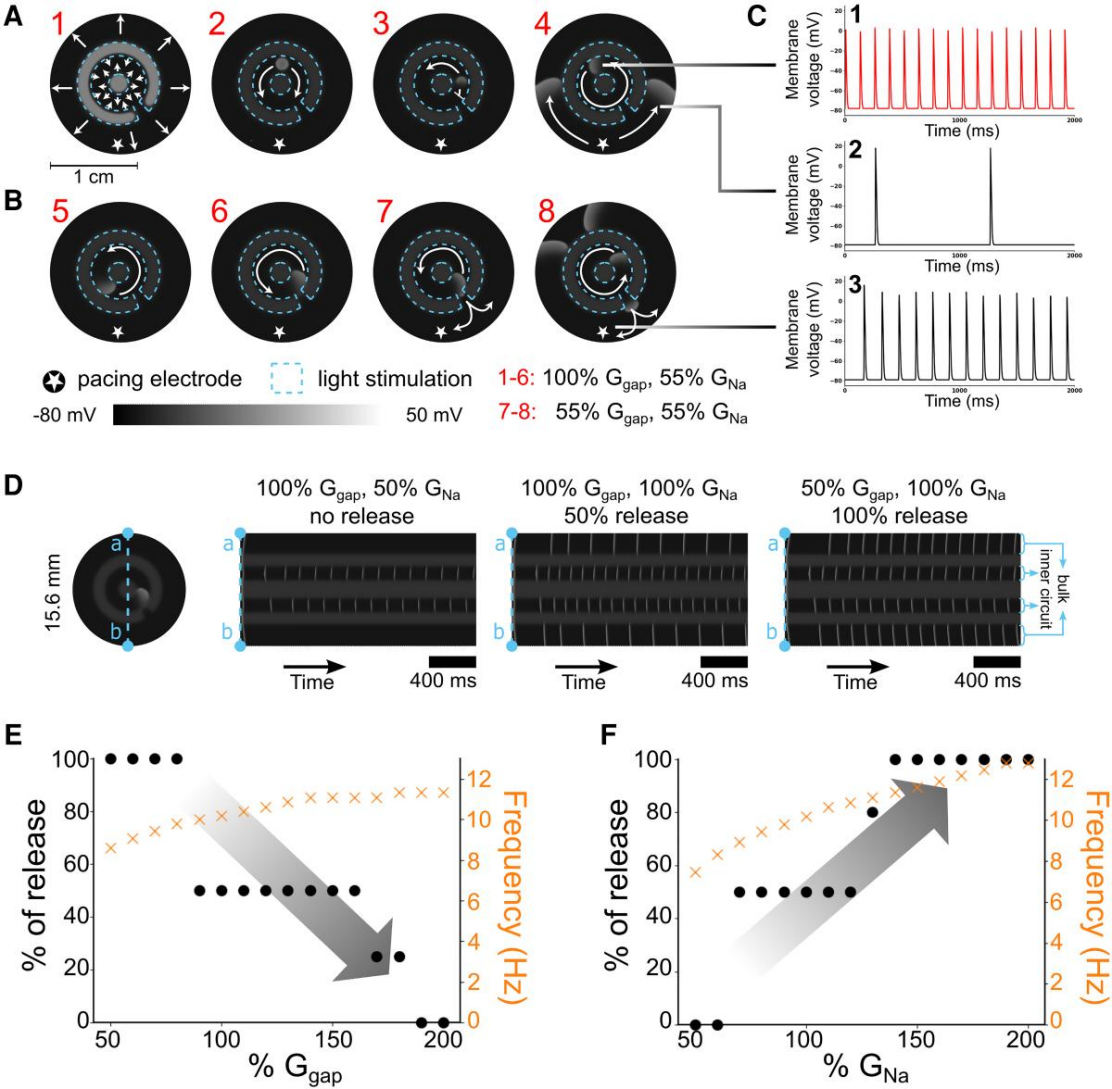
*In silico* realization of trapped re-entry (2D model). (*A*) Procedure to initiate a trapped re-entrant wave. Blue dashed lines demarcate the inner circle and outer ring in which the CatCh channels are activated. Light-coloured areas are depolarized, dark-coloured areas are repolarized. (*B*) Escape of a trapped re-entrant wave following reduction of gap junctional coupling. (*C*) Voltage traces of three selected representative points showing trapped re-entry with SR in the surrounding bulk tissue in A4 and escape of the re-entrant wave through an isthmus in B8. (*D*) Line analysis of the electrical activity through the centre of the circuit (vertical axis) over time (horizontal axis). Different release rhythms are observed depending on the global parameters used in the model (see the signals in the bulk tissue at the top and bottoms of each panel). Scale bar: 400 ms. (*E*) Escape and rotational frequency of trapped waves at different levels of gap junctional coupling (*G*_Na_ = 100%). (*F*) Escape and rotational frequency of trapped waves at different sodium conductances (*G*_gap_ = 100%).

The principle of trapped re-entry was first demonstrated in a simplified controllable system (that could later be realized experimentally): an *in silico* monolayer model of NRAMs^22^ (*Figure 1*, Supplementary material online, *Video V1*). Excitation and conduction block were mimicked by the introduction of a virtual channelrhodopsin into the cells.^23^ When these light-gated cation channels are activated by simulated light, a depolarizing current is generated that makes the membrane potential less negative as long as the light is on, rendering the illuminated regions inexcitable. Consequently, any geometry of structural heterogeneity can be created, e.g. a defined geometry of depolarized tissue containing an isolated conductive circuit, with an isthmus to access it from the outside. The simplest construction of such a geometry is shown in *Figure 1A* and *B*: an isolated circuit between two circular obstacles with an isthmus in the outer ring.

Establishment of trapped re-entry in this isolated circuit involved the following steps:

1. Patterned light pulse to create the circuit of trapped re-entry (*Figure 1A1*),
2. S1 pulse inside the circuit to initiate excitation waves propagating in both directions (*Figure 1A2*),
3. Properly timed S2 pulse, simulating an ectopic pulse, to block counterclockwise wave propagation and initiate re-entry (*Figure 1A3*) at frequencies between 7.46 and 12.82 Hz (*Figure 1C1, D*, *E* and *F* orange data points), and
4. Consolidation of trapped re-entry due to current-to-load mismatch at the isthmus (*Figure 1A4*) under SR (*Figure 1C2*).

Escape of the trapped waves is shown in *Figure 1B*, leading to focal arrhythmia in the bulk tissue (*Figure 1C3*). While such release can be achieved by various means, in this case we globally modified sodium channel and gap junctional conductance (*G*_Na_ and *G*_gap_, respectively).

Globally decreasing *G*_Na_ increases source-sink (re-entry circuit-surrounding bulk tissue) mismatch from inside to outside at the isthmus, and therefore promoted excitation wave trapping. On the other hand, reduction of gap junctional coupling decreases source-sink mismatch, helping the re-entrant waves to escape (*Figure 1B*). Different release patterns were found by adjusting these two parameters, going from total block to release of every re-entrant wave that passed the isthmus. *Figure 1D* shows a narrow slice of the 2D tissue through the centre of the circuit (vertical axis) over time (horizontal axis) in which three different situations are depicted, i.e. no release or block (50% *G*_Na_, 100% *G*_gap_), 50% release (100% *G*_Na_, 100% *G*_gap_), and 100% release (100% *G*_Na_, 50% *G*_gap_).

To get more mechanistic insight into the trapping and releasing of a re-entrant wave, each of the two parameters was varied separately while keeping the other one constant at 100% (*Figure 1E* and *F*, Supplementary material online, *Video V1*). The capture rate, expressed as the percentage of waves able to escape, depended on the extent of source-sink mismatch. Isthmus conduction was impeded by reducing *G*_Na_ and promoted by a decrease in gap junctional coupling. This shows that it is possible to control trapped re-entry by means of changing just two global parameters.

### 3.2 Trapped reentry in a 2D *in vitro* model of NRAMs

**Figure 2:**
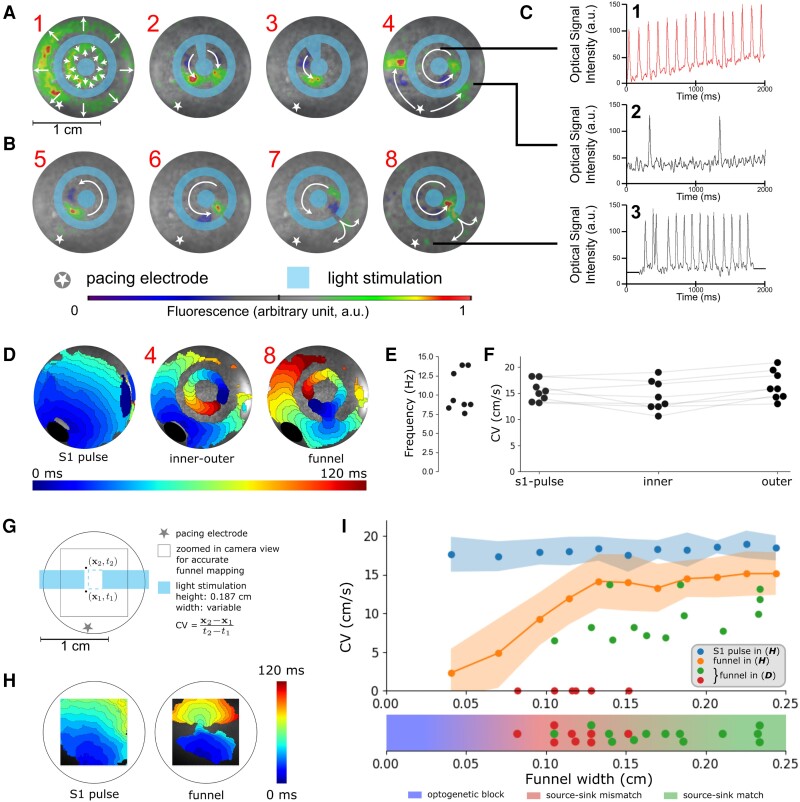
*In vitro* realization of trapped re-entry (2D model). (*A*) Procedure to initiate a trapped re-entrant wave (*n* = 8 biological replicates). The light blue colour shows the illumination pattern used to locally inactivate the tissue. White arrows superimposed on the optical mapping data depict wave propagation. The radially moving waves in A1 occur upon the onset of illumination. (*B*) Opening of the optogenetically isolated circuit of conduction with non-illuminated areas (isthmi) of increasing width, such that escape of the trapped wave can occur. (*C*) Voltage traces at representative points showing trapped re-entry with SR in the surrounding bulk tissue in A4 and escape of the re-entrant wave through an isthmus in B8. (*D*) Activation map of CatCh-expressing NRAM monolayer showing trapped re-entry. (*E*) Rotational frequencies of trapped excitation waves. (*F*) Conduction velocities (CVs) in different regions of the trapped re-entry circuit. The paired Wilcoxon groups signed rank test with *α* = 0.05 showed no significant difference in CV comparing SR and trapped re-entry. (*G*) Scheme showing how funnel CVs were measured in CatCh-expressing NRAM monolayers (*n* = 5 biological replicates). (*H*) Activation maps of CatCh-expressing NRAM monolayer exposed (right) or not exposed (left) to the illumination pattern shown in (*G*). (*I*) CVs as a function of funnel width for the experiments shown in (*A–F*) and in (*G–H*). The red and green dots represent failed and successful release of excitation waves from the re-entrant circuits, respectively. The blue and orange dots correspond to NRAM monolayers without and with an optogenetically imposed rectangular conduction block [i.e. before and after blue light stimulation as shown in (*G*)], respectively. Data of the experiment of (*G–H*) are presented as average ± standard deviation (blue and orange shaded areas, *n* = 5 biological replicates).

Monolayers of CatCh-expressing NRAMs were subjected to patterned illumination to establish conditions for trapped re-entry in a cell culture model (*Figure 2*, Supplementary material online, *Videos V2* and *V3*).

After establishing the geometrical circuit (*Figure 2A1*), a trapped wave was created inside the optogenetically constructed region of isolated conduction by means of an S1S2 protocol (S1: *Figure 2A2*; S2: *Figure 2A3*). This resulted in trapping of a re-entrant wave (*Figure 2A4*) with a frequency between 7.58 and 13.89 Hz inside the isolated region and SR of 1 Hz in the remainder of the monolayer.

Patterned illumination of the CatCh-expressing NRAMs allowed precise control of local source-sinks. By projecting patterns with different isthmus widths, control over entrapment and escape of re-entrant waves could be exerted (*Figure 2B*), which revealed more frequent escapes of re-entrant waves for wider isthmi. For eight NRAM monolayers it was possible to create trapped re-entry with this protocol.

Entrapment and escape of a re-entrant wave was demonstrated by representative optical voltage traces from an NRAM monolayer showing a trapped wave in the re-entry circuit (*Figure 2C1*), SR in the surrounding tissue bulk (*Figure 2C2*) and a focal arrhythmia originating from the re-entry circuit in the bulk tissue (*Figure 2C3*). Because the top and bottom traces had the same frequency, the outer area was paced by the excitations that escaped from the re-entry circuit and no longer by sinus pulses. The re-entrant drivers had a frequency of 10.41 ± 2.65 Hz (*Figure 2E*). Conduction velocities through the monolayer under a regular sinus pulse (15.40 ± 1.99 cm/s) were comparable to the conduction velocities inside (14.43 ± 2.91 cm/s) and outside (16.46 ± 2.78 cm/s) the re-entry circuit (*Figure 2D* and *F*), showing little optogenetic effects on the propagation of excitation waves.

To demonstrate the existence of source-sink mismatch at the funnel of the experimental trapped re-entry circuits, five CatCh-expressing NRAM monolayers were locally illuminated to create a rectangular conduction block interrupted in the middle by a rectangular funnel of varying widths (*Figure 2G*, Supplementary material online, *Video V4*). Activation maps were measured at 1 Hz pacing (*Figure 2H*) and the associated conduction velocities (across the funnel) before and after illumination were plotted against funnel width (*Figure 2I*, blue and orange dots). The two different trapped re-entry regimes (failed and successful escape from *Figure 2D-8*) are simultaneously visualized in the same plot (*Figure 2I*, green and red dots, respectively). Entrapment starts to occur for funnel widths below ∼0.13 cm (*Figure 2I*, lower part) in case of a curved excitation block border (more sink), while excitation waves still pass through funnel widths as small as 0.04 cm (*Figure 2I*, orange dots) when they have a less curved excitation block border (less sink).

### 3.3 Optogenetic vs. fibrotic realization of trapped reentry

**Figure 3:**
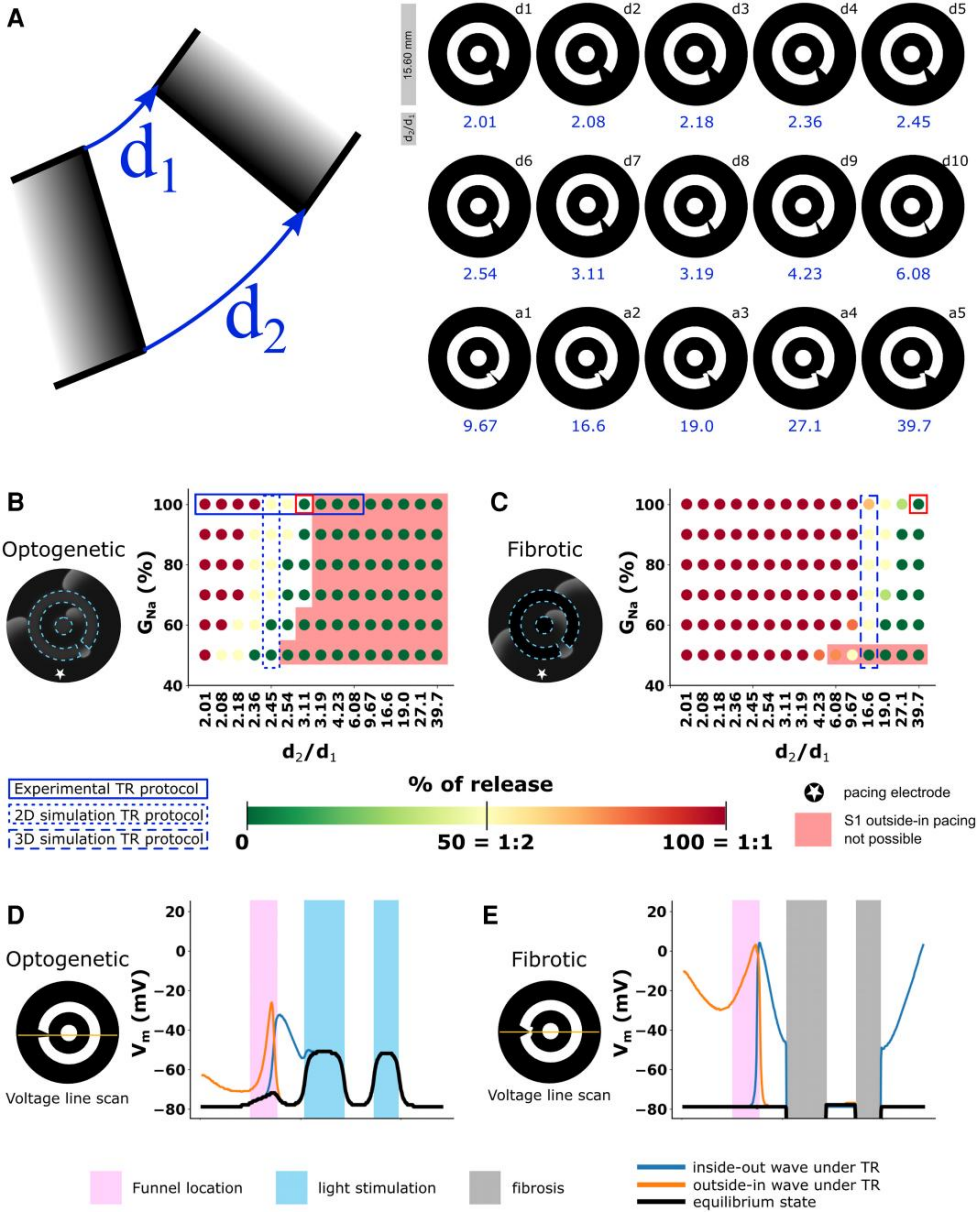
Optogenetic vs. fibrotic realization of trapped re-entry. (*A*) The isthmus of a trapped re-entry (TR) circuit can be characterized by the ratio between its inner and outer width (left). Overview of the 15 different isthmus geometries that were tested with their specific *d*_2_/*d*_1_ ratios (right). (*B*) Release percentage of a re-entrant wave as a function of circuit geometry and *G*_Na_ when the geometry is realized through the establishment of an optogenetic conduction block. (*C*) Release percentage of a re-entrant wave as a function of circuit geometry and *G*_Na_ when the geometry is realized through fibrotic non-conducting regions. (*D*, *E*) Voltage (*V_m_*) line scans through the isthmus under the optogenetically imposed (*D*) and fibrosis-related (*E*) conditions of trapped re-entry red square box outlined in (*B*) and (*C*).

In diseased hearts, dense fibrotic regions are a major cause of conduction block. To show that there is a similarity in behaviour between optogenetically created trapped re-entry circuits and those resulting from fibrosis, 2D simulations were performed (*Figure 3*). For isthmi with different *d*_2_/*d*_1_ (isthmus outer opening length/isthmus inner opening length) ratios (*Figure 3A*), both optogenetic conduction block (*Figure 3B*) and fibrotic conduction block (*Figure 3C*) were simulated. Either light was shone onto optogenetically modified virtual NRAMs in the patterns indicated in *Figure 3A* (*Figure 3B*), or non-conducting cells were added to create the same patterns (*Figure 3C*). The shapes were named d1–d10 for variations mainly in the average isthmus opening length, and a1–a5 for variations mainly in the angle of the isthmus. To show that the formation (only possible in the unshaded areas of *Figure 3B* and *C*), perpetuation and release of trapped re-entrant waves under SR can be observed in both the optogenetic and fibrotic models of trapped re-entry, *G*_Na_ was globally varied. A dependence on pacing frequency was observed for excitation waves to enter the circuit through the funnel (see Supplementary material online, *Figure S3*), with higher frequencies sometimes skipping the circuit altogether (see Supplementary material online, *Video V5*). However, once re-entry is initiated, a transition from 0% towards 100% release is observed when the *d*_2_/*d*_1_ ratio is decreased, but also when *G*_Na_ is increased (*Figure 3B* and *C*). While this phenomenon occurs irrespective of the cause of the conduction block, the specific conditions under which it took place depended on the way (optogenetics or fibrosis) by which the circuit of trapped re-entry was created. Both the optogenetics- and fibrosis-based trapped re-entry circuits displayed an excitable gap inside the circuit, but no resetting or entrainment was observed under external SR pacing at different frequencies when re-entry was ongoing (see Supplementary material online, *Video V6*). The external excitation waves could enter only a part of the funnel (*Figure 3D* and *E*). Electrical coupling between illuminated and non-illuminated cells (see black line in *Figure 3D*) caused funnel widths to be larger for the optogenetic trapped re-entry model compared to the fibrotic one. The results nonetheless indicate that optogenetic modelling of trapped re-entry is valuable and that our experimental set-up is sufficient to capture the essence of the trapped re-entry phenomenon. Our findings also suggest that trapped re-entry could be found in real life under circumstances of dense myocardial fibrosis.

### 3.4 Trapped reentry in a 3D *in silico* model of human atria

**Figure 4:**
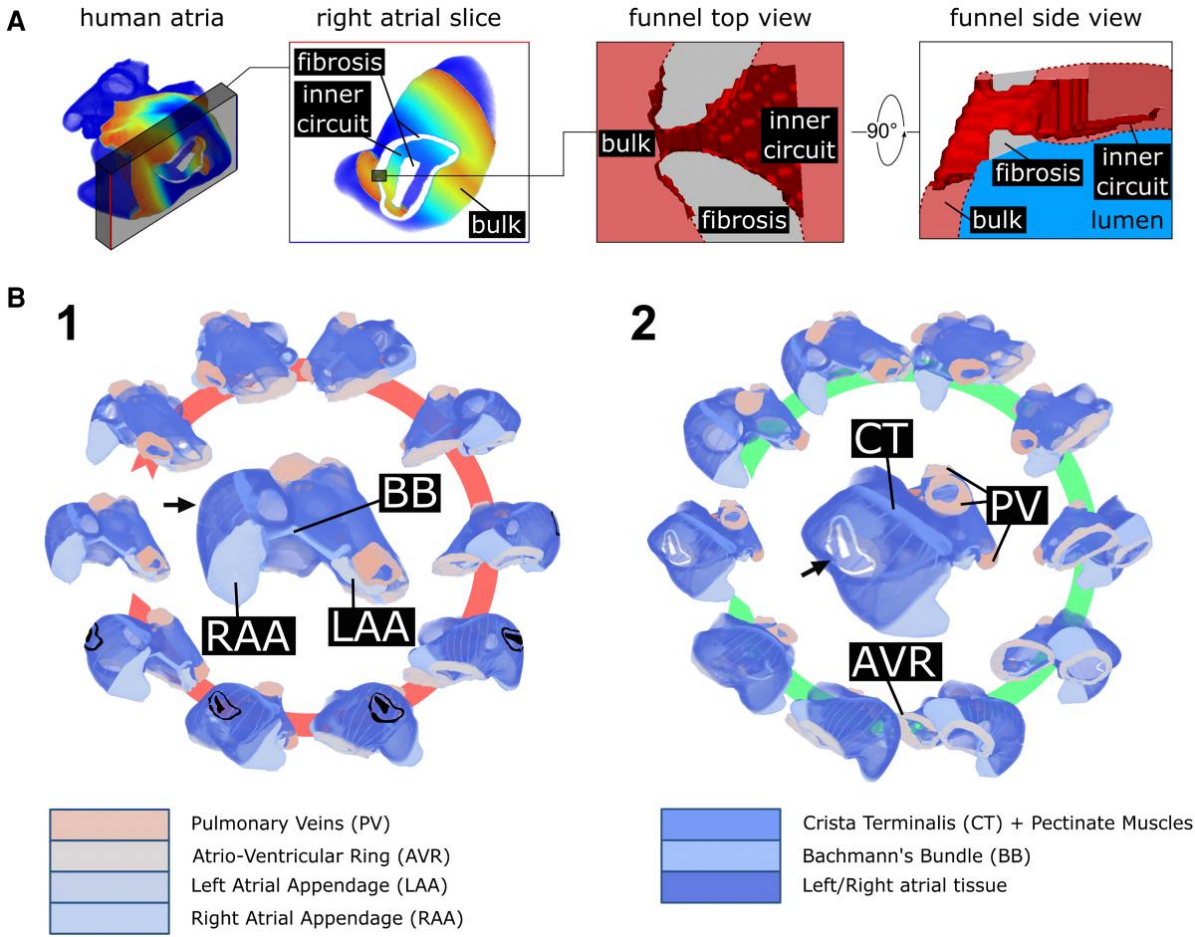
Design of the 3D funnel-shaped isthmus and the trapped re-entry circuit in human atria. (*A*) 3D funnel-shaped isthmus between the circuit of trapped re-entry and the bulk tissue. The funnel is gradually widening towards the inner circuit and has a sharp transition into the bulk of the atria. (*B*) Rotating view of human atria with a circuit of trapped re-entry. The central pictures are enlarged versions of the leftmost pictures in both panels. Atrial structures are indicated by different colours and labels. 1, Middle: anterosuperior view of the atria. For clarity, the circuit of trapped re-entry is indicated in black. When not visible, circuit location is indicated with an arrow. 2, Middle: slightly tilted view relative to (*A*) that better visualizes the circuit of trapped re-entry. Non-conducting tissue is made transparent for better visualization.

To investigate whether trapped re-entry could exist in human atria, 3D simulations were performed in which human atrial geometries were combined with an area of dense fibrosis. Conditions favouring trapped re-entry were created by transmural non-conducting fibrotic regions. A funnel-shaped isthmus was constructed (*Figure 4A*, Supplementary material online, *Video V7*) to connect the bulk of the tissue to the inner trapped re-entry circuit in the right atrial wall of a realistic digital twin of the human atria (*Figure 4B1* and *B2*).

We used three models for human atrial cells, representing different stages of atrial remodelling: (1) the Courtemanche model^25^ for healthy atrial tissue, (2) the AF Courtemanche model^26^ representing paroxysmal AF, and (3) the Loewe model^27^ for chronic AF.

**Figure 5:**
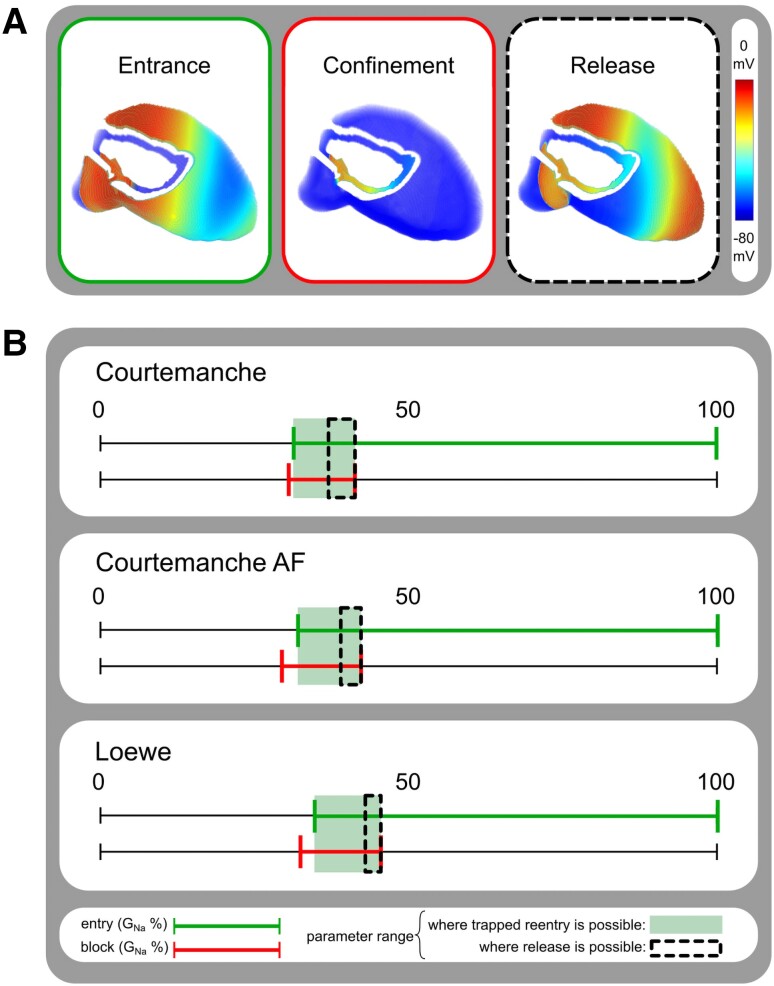
The conditions for trapped re-entry differ between 3D models of healthy and diseased atria. (*A*) Overview of the three key aspects of trapped re-entry: entrance of an excitation wave into an electrically isolated circuit (left), confinement of the trapped wave inside the circuit (middle) and release of the excitation wave from the circuit (right). (*B*) Conditions allowing entrapment and release of a re-entrant wave from an isolated circuit. The upper lines in each panel indicate the range (in green colour) of *G*_Na_ in which the wave can enter the circuit during SR. The lower lines indicate the range (in red colour) of *G*_Na_ in which the wave can be trapped inside the circuit. The overlaps of both lines (light green boxes) mark the regions in which trapped re-entry is possible. The regions in which release of the re-entrant waves is possible by reduction of *G*_gap_ (see Supplementary material online, *Table T1*) are indicated by dashed lines.

For each of these models, we found values of global *G*_Na_ and global *G*_gap_ causing excitation waves to enter the isolated conduction circuit, to be trapped inside this circuit or to be released from the circuit (*Figure 5A*), i.e. conditions that imposed unidirectional block (for entry and trapping) and bidirectional propagation (for escape) at the isthmus.

*Figure 5B* summarizes the results obtained with different values of global *G*_Na_ at 100% *G*_gap_ for each of the three atrial models (for details, see Supplementary material online, *Table T1*). In particular, global *G*_Na_ > 31.4% allowed propagation waves to enter the circuit from the outside for the Courtemanche model of healthy human atrial tissue (green bar). Waves rotating inside the circuit could not exit at global *G*_Na_ < 41.3% and only kept propagating for global *G*_Na_ > 30.6% (red bar). In the intersecting region, excitation waves could enter the circuit but could not escape from it (light green shading). Release of the re-entrant waves could be accomplished by globally lowering *G*_gap_ but was only possible for points inside the green region surrounded by the dashed line. Similar results were obtained for the other two models (*Figure 5B*). The parameter range allowing release of trapped waves appeared to be largely independent of the length of the inner circuit although the escape frequency was higher for the longer re-entrant circuits (see Supplementary material online, *Figure S4*). Moreover, simulations at a higher spatial resolution (i.e. 100 µm) revealed larger parameter regions supporting trapped re-entry (see Supplementary material online, *Figure S5*).

### 3.5 Clinical translation

**Figure 6:**
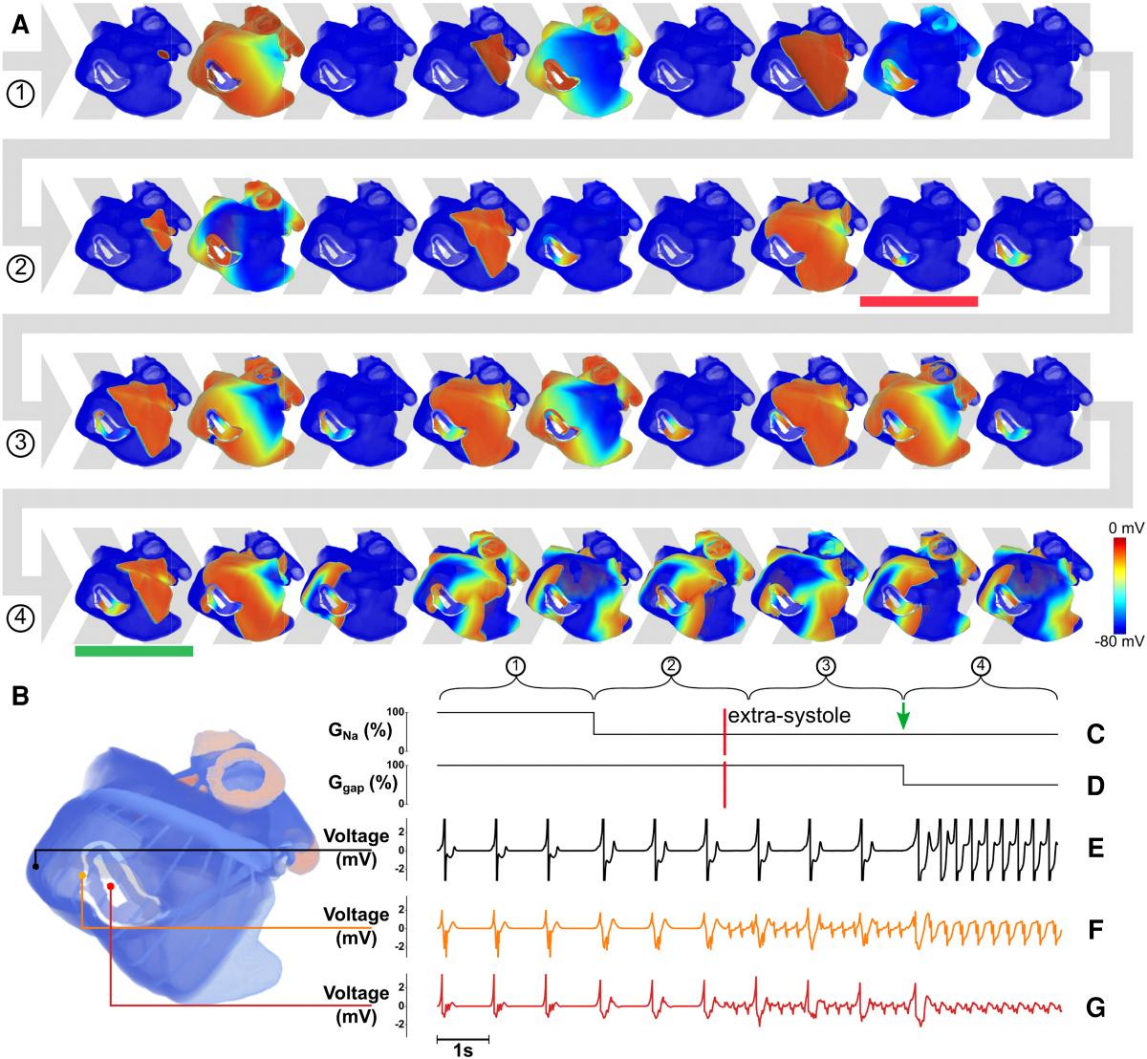
3D realization of trapped re-entry linked to unipolar electrograms. (*A*) Visualization of the steps involved in trapped re-entry through representative voltage maps (3 pictures/s, 12 s in total). The horizontal red bar denotes the moment an extrasystole and subsequent trapping occurs, while the horizontal green bar marks the escape of the trapped excitation wave. (*B*) Enlargement of the human atria with anatomical regions indicated by different colours. (*C*) Relative sodium conductance. (*D*) Relative gap junctional coupling efficiency. (*E*) Unipolar electrogram showing SR and tachyarrhythmia in the bulk atrial tissue. (*F*, *G*) Unipolar electrograms next to the circuit of trapped re-entry showing fractionation during SR.

In a clinical setting, it is currently not feasible to record wave excitation patterns at the same spatial resolution as in simulations or in cardiac monolayer cultures. Therefore, in realistic full atrial simulations (Loewe model) we calculated unipolar epicardial electrograms (*Figure 6* and Supplementary material online, *Figure S6*, *Videos V8* and *V9*) to derive clinically relevant data.

In *Figure 6* and Supplementary material online, *Figure S6*, simulations of trapped re-entry were run in a digital twin of human atria with a locally isolated conduction circuit located in the right atrium (as in *Figure 4*). These simulations spanned 12 s in real-time, which were divided in four sections of 3 s (*Figure 6A* and Supplementary material online, *Figure S6A*, rows 1 through 4) with 3 images per second.

SR was applied from the sinoatrial node at a frequency of 1 Hz for the duration of the simulation. Local re-entry was initiated by timing an extrasystole (S2 pulse; horizontal red bars in *Figure 6A* and Supplementary material online, *Figure S6A*, vertical red lines in *Figure 6C* and *D* and *Figure S6C* and *D*) inside the electrically isolated region after a sinus pulse had entered. However, other initiation mechanisms might exist (see Supplementary material online, *Figure S7B* and *C*).

To obtain trapped re-entry, the active (*G*_Na_) and passive (*G*_gap_) properties of the tissue were changed based on our previous parameter analysis. The timing of these changes slightly differed between *Figure 6* and Supplementary material online, *Figure S6* to illustrate that trapped re-entry can be induced directly (*Figure 6*) as well as after modification of the tissue properties (see Supplementary material online, *Figure S6*). In *Figure 6*, *G*_Na_ was reduced after 3 s, i.e. before the S2 stimulus that trapped a wave. Subsequent reduction of *G*_gap_ (after 9 s) led to escape of the re-entrant wave from the isolated circuit (horizontal green bar in *Figure 6A*, green arrow in *Figure 6B*). In Supplementary material online, *Figure S6*, *G*_Na_ was reduced after 5 s, which is after the S2 stimulus. As a result, the global re-entrant driver that was established in the isolated circuit, became locally trapped after *G*_Na_ reduction. Escape of the re-entrant wave from the isolated circuit again depended on a decrease (in this case after 10 s) of the gap junctional coupling.

When the reduction in *G*_Na_ preceded the S2 pulse that initiated trapped re-entry as in *Figure 6*, no irregular activity was observed through the unipolar electrodes in the bulk of the atria until the gap junctional coupling decreased. Contrarily, when trapped re-entry was accomplished by applying an S2 pulse before decreasing *G*_Na_ (see Supplementary material online, *Figure S6*), two episodes of high-frequency pacing were observed. The first one occurred between the S2 pulse and the reduction in *G*_Na_, while the second one emerged after the decrease of gap junctional coupling.

**Figure 7:**
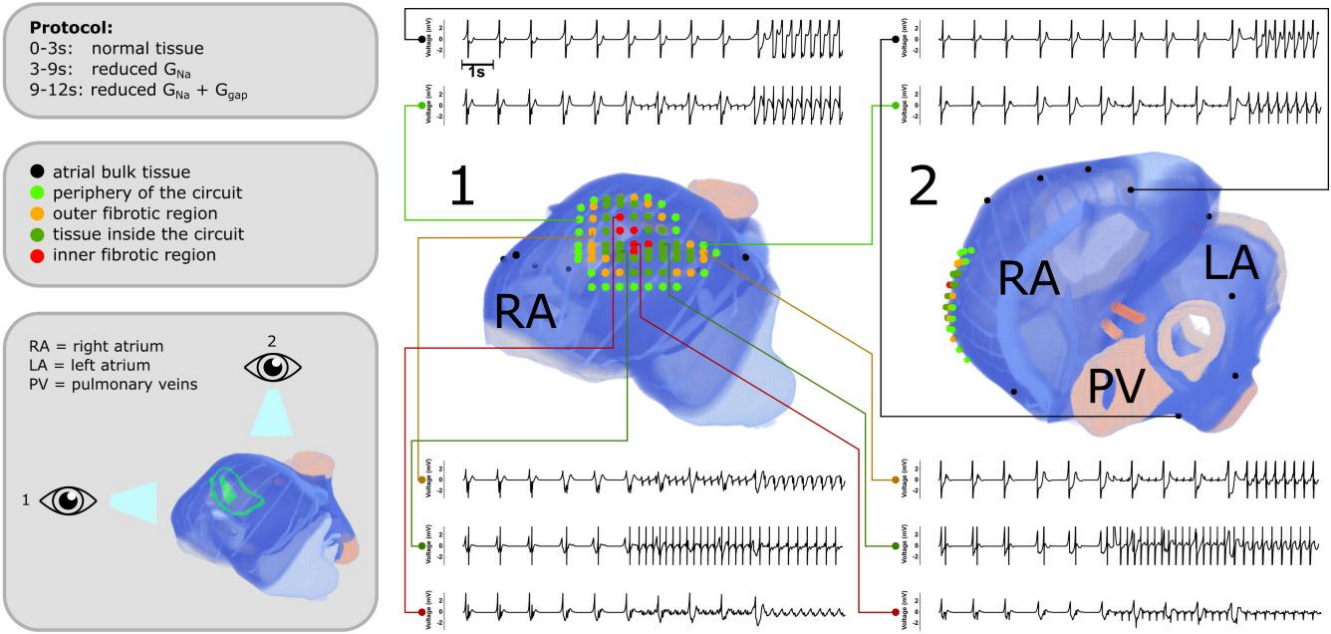
Unipolar electrograms recorded around the circuit of trapped re-entry. A total of 85 epicardial unipolar electrograms were taken, 10 of which are visualized for the process shown in *Figure 6* with their location indicated on two different views of the atria (1, right lateral view; 2, superior view). Black: atrial bulk tissue, light green: periphery of the circuit, orange: outer fibrotic region, dark green: tissue inside the circuit, and red: inner fibrotic region.

For both simulations, epicardial unipolar electrograms were taken at 85 different locations in the atria (*Figure 7*, which is based on the simulations in *Figure 6*). Fundamental differences can be seen between electrograms recorded close to and far away from the circuit of trapped re-entry. Far away from the dense fibrotic region, no influence of the trapped wave was seen (1 Hz signal) until the sudden onset of a fast pacing rhythm of 3.3 Hz after the reduction of gap junctional coupling. However, in a small region around the circuit of trapped re-entry, fractionated atrial electrograms were observed as soon as trapped re-entry was initiated.

## 4. Discussion

The concept of trapped re-entry relies on the temporal electrical isolation of a small region of excitable cardiac tissue in which re-entrant wave propagation can be established. Highly fibrotic regions are favourable for the establishment of re-entrant arrhythmias due to their many narrow conduction pathways. Trapped re-entry can be explained by combining the theories behind impulse propagation block due to abrupt tissue expansion^30^ and impulse transmission at narrow conduction pathways due to partial cellular uncoupling.^20^ A circuit of trapped re-entry thus typically consists of an electrically isolated region of excitable tissue connected to the bulk tissue through a narrow opening. This isthmus allows control over the excitation waves entering or leaving the re-entry circuit through dynamic changes in electrophysiological tissue properties. Different realizations of such a circuit were demonstrated in both *in silico* and *in vitro* models. These models all contained two distinct regions of conduction block, the first one being the core of the circuit, and the second one being a ring around the excitable tissue in the circuit, with an opening (the isthmus) allowing entrance and escape of excitation waves. The isthmus that connects the isolated region to the bulk of the tissue can be manipulated to influence source-sink relationships. This can be achieved by changing the active and passive electrical properties of the tissue^31^ or by altering the geometrical properties of the isthmus through modifications of the inexcitable region that borders the circuit^32^ (*Figure 3*).

In our two 2D models of trapped re-entry, fibrosis was mimicked by means of optogenetically induced conduction block. Especially for establishing trapped re-entry *in vitro*, the optogenetic approach was chosen since this technique allows full control over the parameters that influence source-sink relationships. Another approach might be to use palmitoleic acid or other uncouplers to reduce gap junctional coupling.^20^ This technique would be closer to the 3D *in silico* realization of the studied phenomenon and better mimic what might be happening in ageing human hearts. This would require that the cells are seeded in specific patterns to create funnels of the desired shape, which is technically challenging. The optogenetic approach circumvents this problem. It should be noted, however, that the boundary of an optogenetically generated region of conduction block behaves differently from the boundary of a conduction-blocking fibrotic region. Due to its influence on neighbouring cells, optogenetic depolarization resembles features of cardiac myofibrosis, which can even be a source of ectopic foci.^33,34^ Nevertheless, the similarity in behaviour between optogenetically created circuits of trapped re-entry in virtual and actual NRAM monolayers and circuits of trapped re-entry delimited by fibrotic areas in virtual NRAM monolayers validates the modelling of trapped re-entry using optogenetics (*Figure 3*). The monolayer studies further demonstrated that a tachyarrhythmia can exist locally with SR prevailing in the bulk of the monolayer and that this latent arrhythmic source can be converted into an active driver with global impact.

We have shown that this latent arrhythmic source is a phenomenon that can occur in atria containing cardiomyocytes with differently shaped action potentials representing healthy or electrically remodelled cells (see Supplementary material online, *Figure S4*). When combining the results obtained with different circuits of trapped re-entry and different computational cell models, several conclusions can be drawn.

1. For each mathematical/computational model, the *G*_Na_–*G*_gap_ parameter range allowing escape of trapped waves is similar for different-sized circuits (see Supplementary material online, *Figure S4*). However, despite circuit length independence, it does depend on the geometry of the funnel that connects the re-entrant circuit to the bulk of the atria (*Figure 3*).
2. The highest value of *G*_Na_ at which escape of trapped waves can occur in our 3D models increases with the degree of tissue remodelling (*Figure 5*).
3. Funnels with higher *d*_2_/*d*_1_ ratios allow escape of trapped waves at higher values of *G*_Na_ (*Figure 3*).
4. The 3D computational model of healthy atrial tissue did not allow the establishment of small circuits of trapped re-entry in contrast to the 3D models of diseased atrial tissue with a shortened action potential duration.
5. When the wavelength of the trapped wave is close to the boundary length of the inner obstacle, the re-entrant wave will either not escape or escape intermittently, e.g. during every second or third rotation (see Supplementary material online, *Figure S4*).
6. At least two different ways exist to arrive in the trapped re-entry regime. (1) A drop in *G*_Na_ to a fixed value (e.g. 45%), followed by atrial remodelling. (2) A gradual decrease of *G*_Na_ without a change (within a particular model) of the atrial myocyte properties. A combination of both these scenarios might also occur.
7. For the simulations in human atria, the changes in sodium and gap junctional conductance are in the range of those observed experimentally and computationally, in association with acute myocardial infarction, ischaemia, commotio cordis, loss-of-function mutations and aging,^35^ i.e. an up to 97% reduction of *G*_Na_ ^36–38^ and an up to 55% decrease of *G*_gap_.^37,39^

In our simulation studies, we looked exclusively at changes in two specific global tissue properties (*G*_Na_ and *G*_gap_) to pinpoint trapped re-entry to a specific cause. We also purposefully chose a large cross-sectional area and spatial resolution in our 3D model to exclude any discretization effects, enabling us to ascribe the observed trapping and escape effects solely to these two global parameters. However, besides *G*_Na_ and gap junctional coupling, there exist other source-sink modulators like changes in extracellular sodium concentration, membrane resistance or threshold potential (for example due to a change in *I*_K1_ conductance), fibrosis and tissue geometry. These changes do not have to be global, but can be local.

The large cross-sectional area (and associated large re-entrant circuit) that was used in our 3D model differs from previous *in silico* studies showing that lone sources of re-entry might exist in dense fibrotic regions close to the percolation threshold. These studies^40,41^ revealed ectopic activity with small minimal cross-sectional areas of the 1 pixel funnel opening to create unidirectional conduction block. As a consequence of the large re-entrant circuit, our electrograms only show continuous and monomorphic activity, while the random nature of the fibrotic tissue in the aforementioned studies^40,41^ results in more fractionated electrograms, hinting at the possible existence of more severe cases of AF than the focal arrhythmias described by us (e.g. instant AF). Trapped re-entry also represents just one example of a latent arrhythmia as one may assume that trapped ectopic foci can exist, in which a central ectopic source is surrounded by an outer fibrotic ring with an isthmus. The trapped re-entry phenomenon might also have a link with sudden cardiac death due to ventricular tachyarrhythmias caused by the escape of trapped excitation waves. Altogether the probability of observing the phenomenon of trapped re-entry in real life is probably larger than the current results suggest by implementing only global changes, and remains subject for future investigation.

Resembling certain features of our ‘trapped re-entry’ concept *in vivo*, it was shown both in dogs^42^ and in humans^43^ that adenosine and tetrodotoxin treatment caused so-called ‘exit block’ of micro-re-entries formed within the sinoatrial node. Micro-re-entry has been shown to exist in the atrial wall as well,^9^ showing the potential for translational exploration. In favour of such translational exploration, there exists clinical evidence of continuous abnormal electrical behaviour under SR.^44^ So-called AF nests^11,45^ represent regions with high-frequency electrical activity under SR. These highly resonant, localized atrial sites may produce CFAEs during AF.^12^ While AF nests and CFAE regions not always overlap,^46^ characteristics of both are present in trapped re-entry circuits. In a study of six individuals without and 34 patients with idiopathic drug-refractory paroxysmal or persistent AF, numerous AF nests were found in all AF patients and in 1/6 of the control subjects. AF induction was possible in the latter individual despite having no history of spontaneous AF, but not in the other five control subjects.^11^ Ablation of these AF nests together with pulmonary vein isolation (PVI) resulted in a lower AF recurrence rate than PVI alone.^45^ Another recent study provided an additional indication of trapped re-entry in the form of scar-related small re-entries (SRSRs).^47^ These SRSRs occurred in regions showing dense fibrosis and gave rise to focal arrhythmias, which could be terminated through focal ablation.

The present study was designed to explore the concept of trapped re-entry in a reproducible and standardized manner for an in-depth quantitative assessment of the parameters involved. With our experimental approach based on combined *in vitro* and *in silico* modelling, we were able to provide proof for the existence of trapped re-entry and detailed insight into the conditions under which such arrhythmic activity could be developed, trapped and released. Given the complex nature of trapped re-entry, we expect that this insight will help the exploration of trapped re-entry *in vivo*, i.e. what to look for under which conditions. Such studies could benefit from the recent progress in cardiac tissue printing by creating (human) atrial structures with predefined fibrotic regions to determine the spatial requirements and electrophysiological conditions for trapped re-entry and release of the trapped wave to occur. The shape of these fibrotic regions could be guided by imaging data from fibrotic arrhythmic atria, allowing the functional assessment of trapped re-entry in a translationally relevant context. In addition, dedicated animal models of AF with dense atrial fibrosis could be subjected to combined high-resolution cardiac imaging (of myocardial fibrosis) and electroanatomical mapping for *in vivo* investigation of trapped re-entry. The mapping catheters should be able to capture a trapped re-entry circuit on a single catheter when it is kept in a single position for several sinus beats. When trying to find small trapped re-entry circuits with this catheter, researchers should be extra careful to check whether there is pacing capture before labelling an area as scar tissue. This measure should be taken because a trapped re-entry circuit might be missed (see Supplementary material online, *Figure S3*) when atrial or coronary sinus pacing is performed at frequencies above SR.^48^ Therefore, electroanatomical maps should be made at several pacing frequencies before concluding that a certain region is non-conductive. In terms of a first clinical exploration in patients, retrospective studies could be instrumental by focusing on reanalysis of existing high-resolution mapping data of fibrotic atria from AF patients, especially when obtained during SR. Such reanalysis may result in reinterpretation of localized aberrant electrical signals surrounded by normal SR signals, especially when available imaging data allows colocalization with enabling structural features at a resolution high enough to consider trapped re-entry as an alternative explanation. The outcome of these studies may set the stage for detailed prospective research into trapped re-entry to assess its features, prognostic value and clinical relevance also in relation to interventional outcome. At this stage, patients should be carefully selected based on the knowledge acquired in the aforementioned research. As of yet, without this knowledge, the combination of AF nests, SRSRs and fractionation during SR suggests that latent arrhythmias could exist during SR and may hence be of additional value for patient selection. This notion also strengthens our idea that AF recurrence could be prevented through early recognition and treatment of trapped re-entry circuits by combining high-resolution electroanatomical mapping and cardiac imaging after appropriate patient selection. Future research should investigate this possibility, for which our present study may be an incentive.

##  

**Translational perspective**

Abnormal electrical behaviour under sinus rhythm has been found in the atria in the form of so-called complex fractionated atrial electrograms and atrial fibrillation nests. We show that this behaviour could be a sign of an underlying ‘sleeping’ arrhythmia, here referred to as trapped re-entry, that can ‘wake up’ and cause a tachyarrhythmia in the whole atria. With this new insight, we aim to trigger the active search for trapped re-entry circuits in patients, to incite discussion among cardiac electrophysiologists about the clinical relevance of (awakening) dormant arrhythmias, and to fuel the search for improvements in arrhythmia treatment.

## Supplementary material

Supplementary material is available at *Cardiovascular Research* online.

## Authors’ contributions

Conceptualization: K.Z., D.A.P.; Methodology: T.D.C., A.S.T., I.F., C.I.B., A.A.R., B.L.d.O.; Investigation: T.D.C., A.S.T., I.F.; Formal Analysis: T.D.C.; Resources: A.A.F.d.V., D.A.P.; Writing—original draft: T.D.C., D.L.Y., A.V.P., A.A.F.d.V., D.A.P.; Writing—review & editing: T.D.C., S.A.T., A.A.F.d.V., D.A.P.

## Supplementary Material

cvad179_Supplementary_Data

## Data Availability

Raw data are available from the corresponding authors upon reasonable request.
