# Supplementary material for: ‘Trapped re-entry’ as source of acute focal atrial arrhythmias
Source: Cardiovasc Res. 2023 Dec 4;120(3):249–61. doi: 10.1093/cvr/cvad179 (PMC10939464; doi:10.1093/cvr/cvad179)
Supplement: cvad179_Supplementary_Data [file cvad179_supplementary_data.zip › MajorResourcesTable_TR_CVR_23_08_21.docx]

**Major Resources Table**

**DNA/cDNA Clones**

| **Clone Name** | **Sequence** | **Source / Repository** | **Persistent ID / URL** |
| --- | --- | --- | --- |
| CatCh | atggactatggcggcgctttgtctgccgtcggacgcgaacttttgttcgttactaatcctgtggtggtgaacgggtccgtcctggtccctgaggatcaatgttactgtgccggatggattgaatctcgcggcacgaacggcgctcagaccgcgtcaaatgtcctgcagtggcttgcagcaggattcagcattttgctgctgatgttctatgcctaccaaacctggaaatctacatgcggctgggaggagatctatgtgtgcgccattgaaatggttaaggtgattctcgagttcttttttgagtttaagaatccctctatgctctaccttgccacaggacaccgggtgcagtggctgcgctatgcagagtggctgctcacttgtcctgtcatctgtatccacctgagcaacctcaccggcctgagcaacgactacagcaggagaaccatgggactccttgtctcagacatcgggactatcgtgtggggggctaccagcgccatggcaaccggctatgttaaagtcatcttcttttgtcttggattgtgctatggcgcgaacacattttttcacgccgccaaagcatatatcgagggttatcatactgtgccaaagggtcggtgccgccaggtcgtgaccggcatggcatggctgtttttcgtgagctggggtatgttcccaattctcttcattttggggcccgaaggttttggcgtcctgagcgtctatggctccaccgtaggtcacacgattattgatctgatgagtaaaaattgttgggggttgttgggacactacctgcgcgtcctgatccacgagcacatattgattcacggagatatccgcaaaaccaccaaactgaacatcggcggaacggagatcgaggtcgagactctcgtcgaagacgaagccgaggccggagccgtgccagcggccgccaccatggtgagcaagggcgaggagctgttcaccggggtggtgcccatcctggtcgagctggacggcgacgtaaacggccacaagttcagcgtgtccggcgagggcgagggcgatgccacctacggcaagctgaccctgaagttcatctgcaccaccggcaagctgcccgtgccctggcccaccctcgtgaccaccttcggctacggcctgcagtgcttcgcccgctaccccgaccacatgaagcagcacgacttcttcaagtccgccatgcccgaaggctacgtccaggagcgcaccatcttcttcaaggacgacggcaactacaagacccgcgccgaggtgaagttcgagggcgacaccctggtgaaccgcatcgagctgaagggcatcgacttcaaggaggacggcaacatcctggggcacaagctggagtacaactacaacagccacaacgtctatatcatggccgacaagcagaagaacggcatcaaggtgaacttcaagatccgccacaacatcgaggacggcagcgtgcagctcgccgaccactaccagcagaacacccccatcggcgacggccccgtgctgctgcccgacaaccactacctgagctaccagtccgccctgagcaaagaccccaacgagaagcgcgatcacatggtcctgctggagttcgtgaccgccgccgggatcactctcggcatggacgagctgtacaagtaa | doi: [10.1093/cvr/cvu179](https://doi.org/10.1093/cvr/cvu179) |  |

**Animals (in vitro studies)**

| **Species** | **Vendor or Source** | **Background Strain** | **Sex** | **Persistent ID / URL** |
| --- | --- | --- | --- | --- |
| Rat | Charles River | Wistar | M/F | RRID:RGD_737929 |

**Cultured Cells**

| **Name** | **Vendor or Source** | **Sex (F, M, or unknown)** | **Persistent ID / URL** |
| --- | --- | --- | --- |
| Primary cultured neonatal rat atrial myocytes | Isolated in-house | both |  |

**Data & Code Availability**

| **Description** | **Source / Repository** | **Persistent ID / URL** |
| --- | --- | --- |
| Software, algorithm: graphics processing unit-usable code | doi:[10.1152/ajpheart.00109.2006](https://doi.org/10.1152/ajpheart.00109.2006) , doi:[10.1038/srep20835](https://doi.org/10.1038/srep20835) |  |
| Python 2.7 |  |  |
| ParaView |  |  |
| BrainVision Analyzer 1101 software | Brainvision, Tokyo, Japan |  |
| PolyLite software | Mightex Systems, Toronto, ON |  |

**Other**

| **Description** | **Source / Repository** | **Persistent ID / URL** |
| --- | --- | --- |
| Ryzen Threadripper 2950X 16-core | AMD |  |
| GeForce RTX 2080Ti | NVIDIA |  |
| 450 U/ml collagenase type I | Worthington, Lakewood, NJ |  |
| 18.75 Kunitz/ml DNase I | Sigma-Aldrich, St.Louis, MO |  |
| Primaria culture dishes | BectonDickinson, Breda, the Netherlands |  |
| round glass coverslips (d=15mm) | GerhardMenzel, Braunschweig, Germany |  |
| fibronectin (100 μg/ml) | Sigma-Aldrich, St.Louis, MO |  |
| Mitomycin-C (10 μg/ml) | Sigma-Aldrich, St.Louis, MO |  |
| voltage-sensitive dye di-4-ANBDQBS (52.5 μM) | ITK diagnostics, Uithoorn, the Netherlands |  |
| MiCAM ULTIMA-L imaging system | SciMedia, CostaMesa, CA |  |
| patterned illumination device (Polygon400) | Mightex Systems, Toronto, ON |  |
| 470 nm, high-power collimator LED source (50 W) | Mightex Systems, Toronto, ON |  |
