# Supplementary material for: ‘Trapped re-entry’ as source of acute focal atrial arrhythmias
Source: Cardiovasc Res. 2023 Dec 4;120(3):249–61. doi: 10.1093/cvr/cvad179 (PMC10939464; doi:10.1093/cvr/cvad179)
Supplement: cvad179_Supplementary_Data [file cvad179_supplementary_data.zip › suppl_TR_CVR_23_08_21.docx]

**Supplementary Data**

**“Trapped reentry” as source of acute focal atrial arrhythmias**

**Authors:**

Tim De Coster^1^, Alexander S. Teplenin^1^, Iolanda Feola^1^, Cindy I. Bart^1^, Arti A. Ramkisoensing^1^, Bram L. den Ouden^1^, Dirk L. Ypey^1^, Serge A. Trines^1^, Alexander V. Panfilov^1,2,3,4^, Katja Zeppenfeld^1^, Antoine A.F. de Vries^1^, Daniël A. Pijnappels^1^

**Affiliations:**

^1^Laboratory of Experimental Cardiology, Department of Cardiology, Heart Lung Centre Leiden, Leiden University Medical Center, 2333 ZA Leiden, the Netherlands

^2^Ghent University, 9000 Ghent, Belgium

^3^Ural Federal University, Biomed Laboratory, 620002 Ekaterinburg, Russia;

^4^World-Class Research Center “Digital Biodesign and Personalized Healthcare,” I. M. Sechenov First Moscow State Medical University, 119146 Moscow, Russia

**Corresponding authors:**

Correspondence and requests for materials should be addressed to DAP (D.A.Pijnappels@lumc.nl) or to TDC (T.J.C.De_Coster@lumc.nl).

**SUPPLEMENTARY FIGURES/TABLES**

**
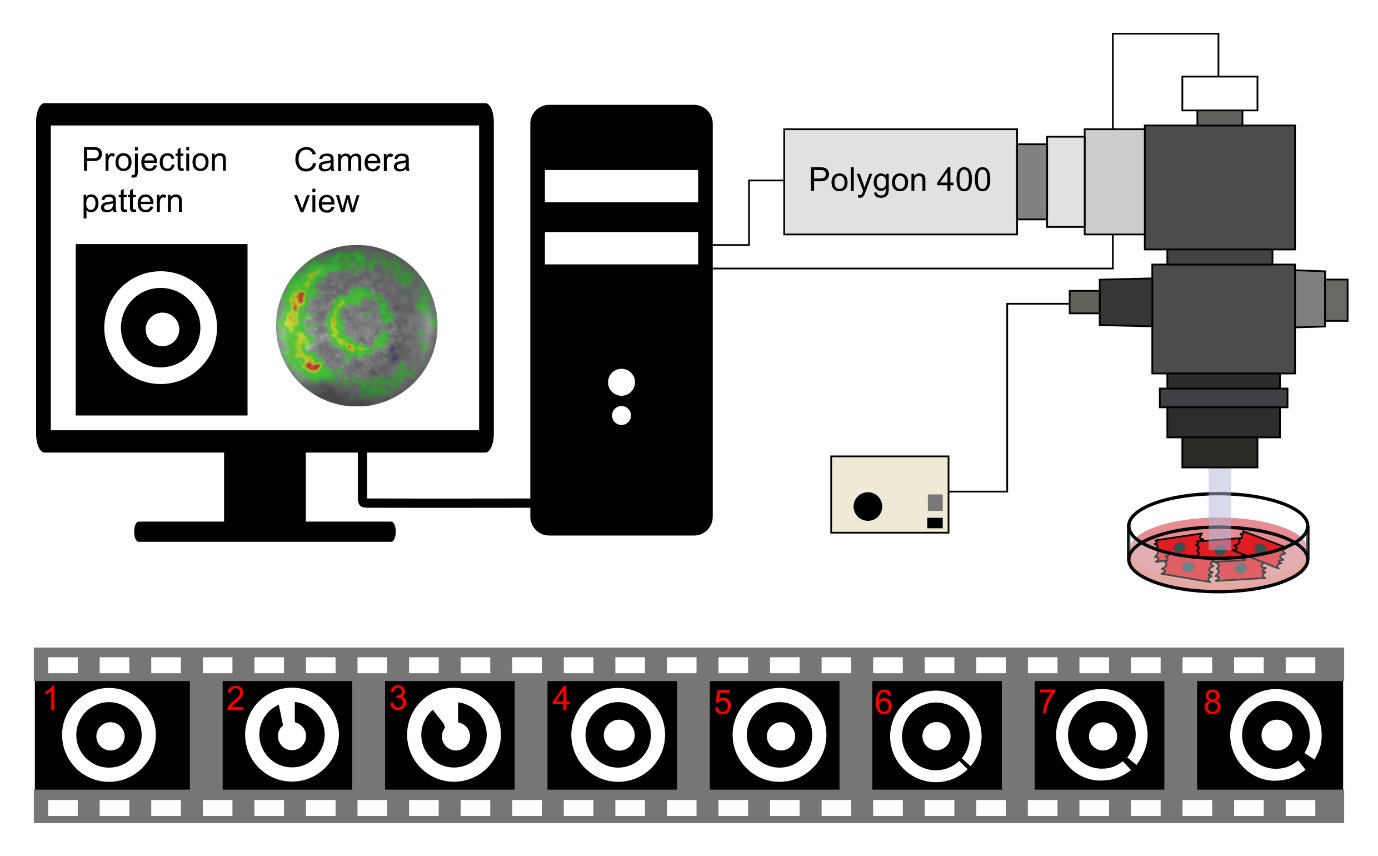
**

**Figure S1. Experimental set-up.** Schematic view of the optical mapping set-up used to carry out the *in vitro* experiments. The patterned illuminator (Polygon 400) shines light according to the patterns depicted in the eight movie frames to first trap and subsequently release a reentrant wave.


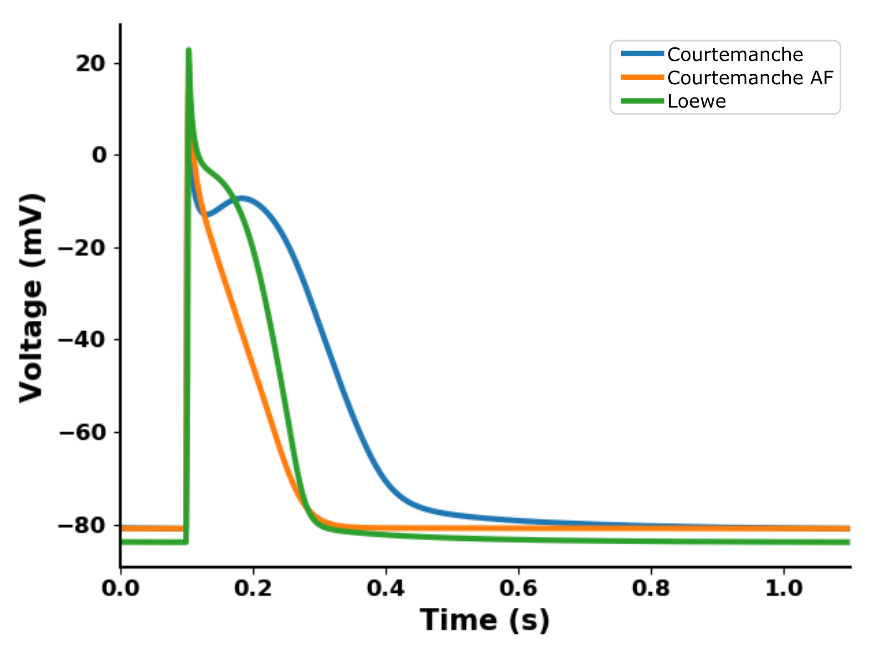
**Figure S2. Atrial action potentials.** Action potential shapes of the three different atrial models that were used after 50 initiation pulses at 1 Hz. The Courtemanche AF and Loewe models represent early and late stages of atrial electrical remodeling, respectively.


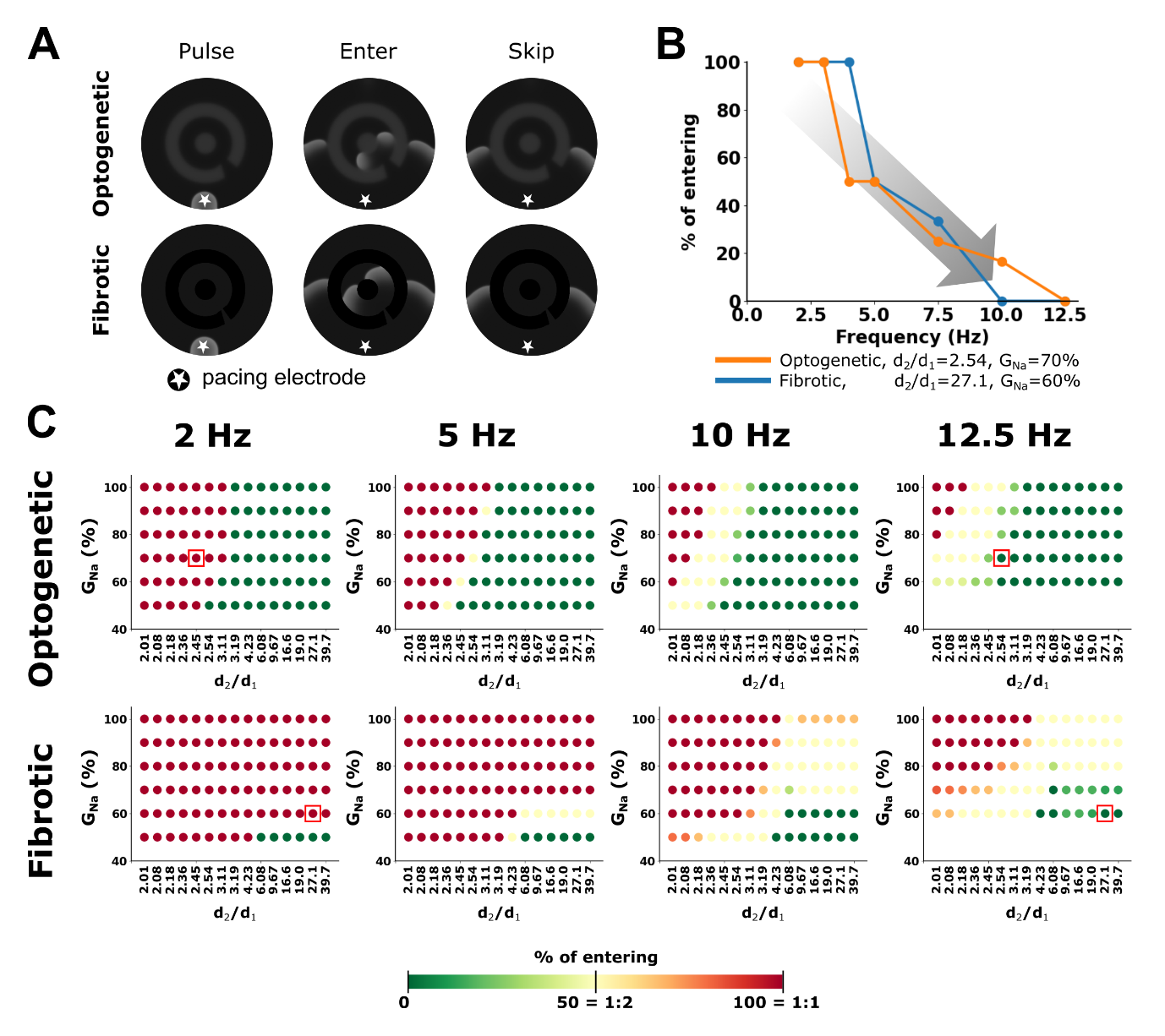


**Figure S3. Sinus wave frequency effects on the entrance of an excitation wave in the optogenetic and fibrotic virtual models of trapped reentry. A**, Location of the sinus pulse (star) and visualization of its two possible outcomes: entry or skipping of the circuit using the conditions red outlined in (**C**). **B**, The percentage of sinus waves that enters the circuit decreases with increasing pulsing frequency. **C**, The percentage of sinus waves that enters the circuit as function of the funnel properties (shown in Figure 3) and sodium conductance (G_Na_).


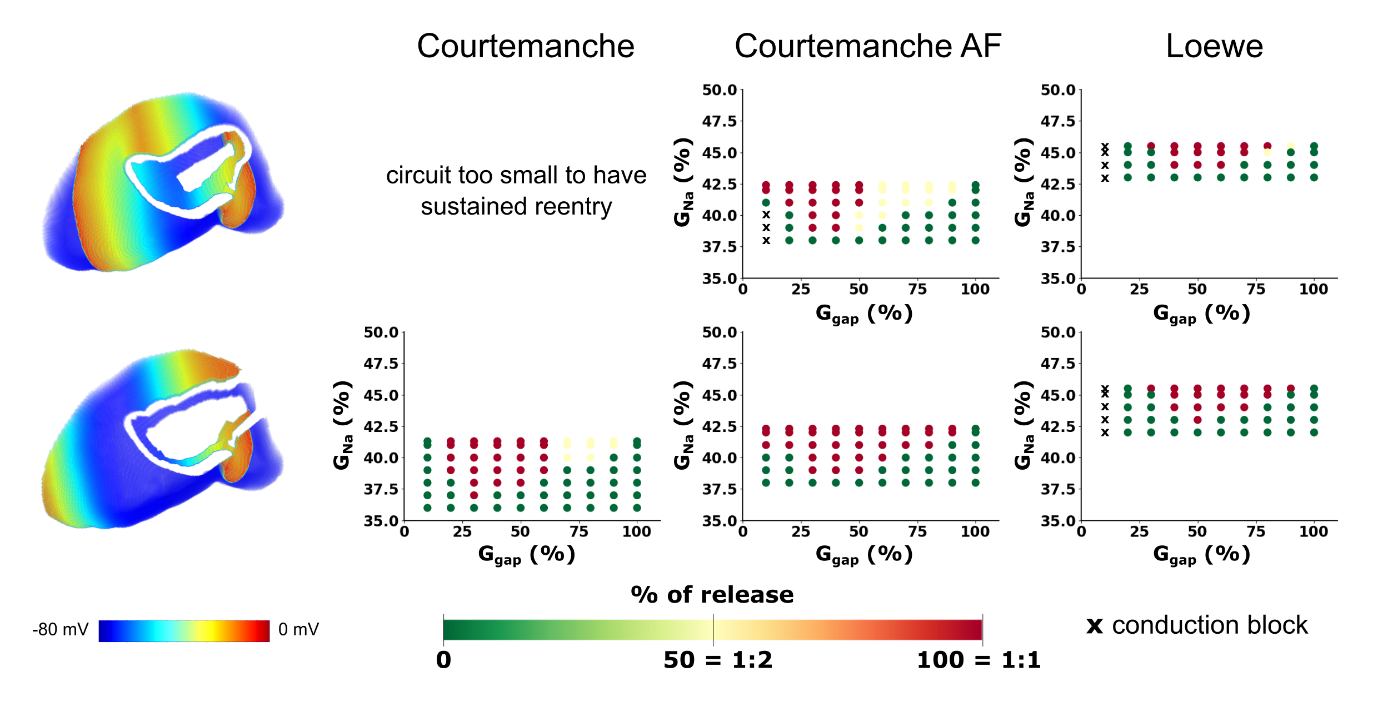
**Figure S4. Overview of sodium and gap junctional conductances allowing trapping and release of excitation waves in three different 3D models of human atria.** For both trapped reentry circuits depicted at the left, results are shown for the Courtemanche, Courtemanche AF and Loewe model.


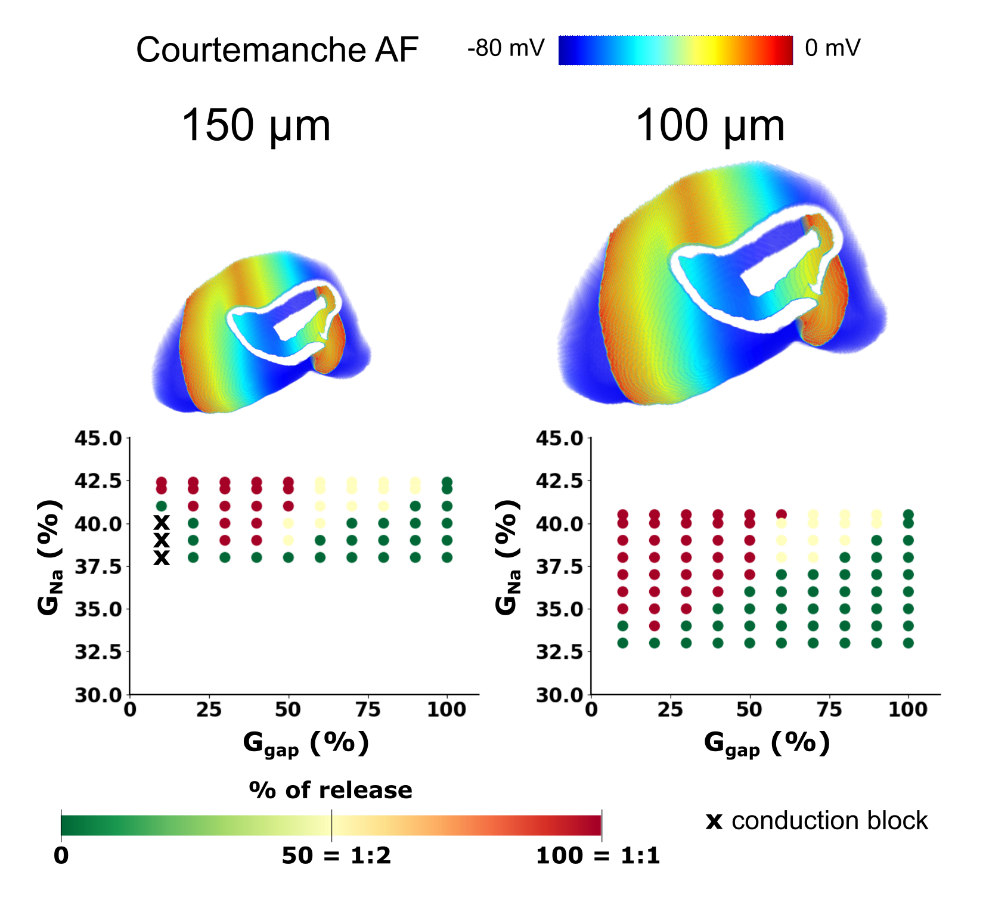
**Figure S5. Conditions allowing trapping and release of excitation waves in 3D models of human atria depend on the resolution with which simulations are run.** For a fixed trapped reentry circuit, results are shown of simulations ran at 150 and 100 µm spatial resolution.

**
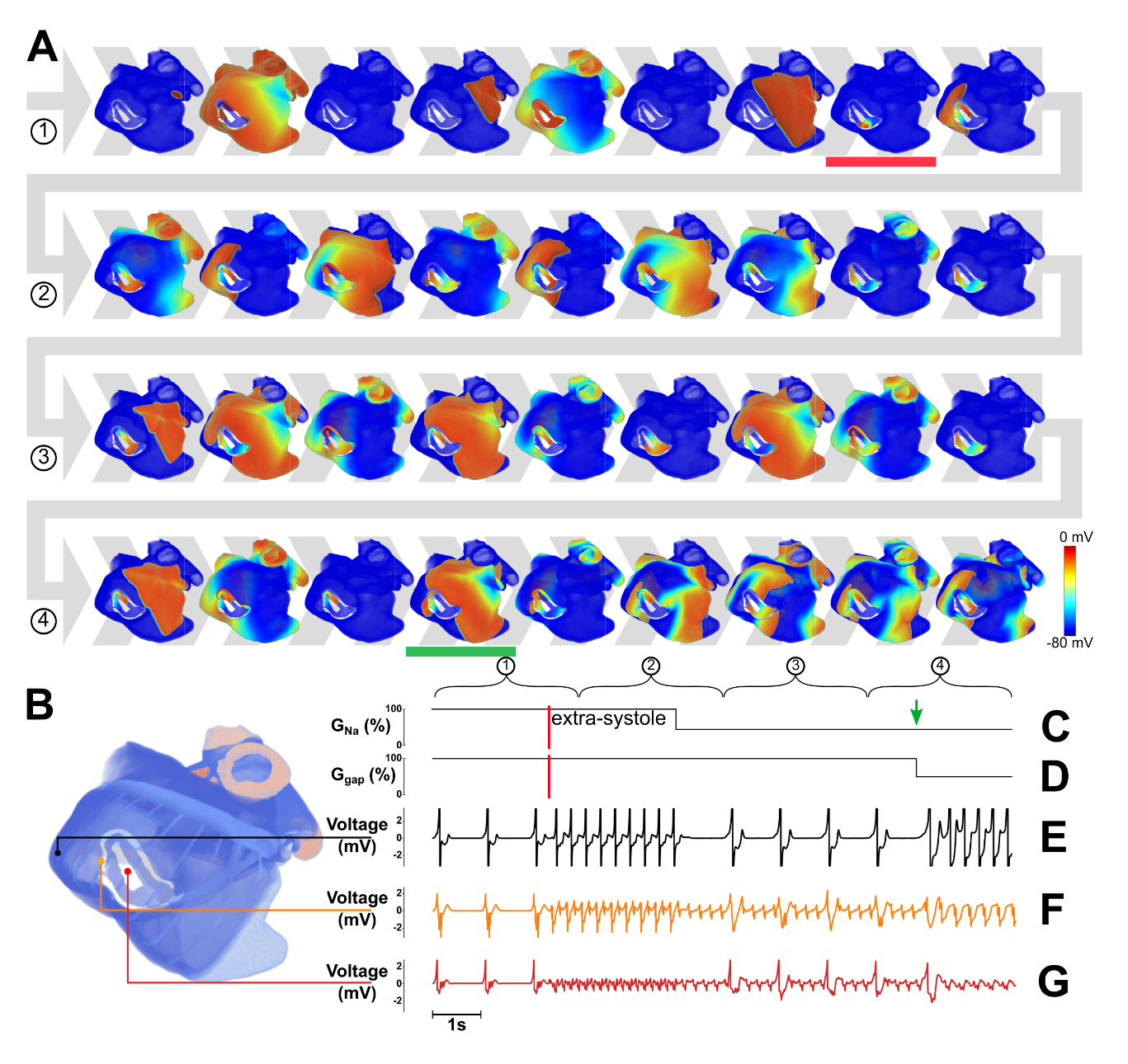
Figure S6. Alternative 3D realization of trapped reentry linked to unipolar electrograms.** **A**, Visualization, by representative voltage maps, of the steps involved in trapped reentry (3 pictures/second, 12 seconds in total). The horizontal red bar denotes the moment an extrasystole occurs, while the horizontal green bar marks the escape of the trapped excitation wave. Trapping occurs when G_Na_ decreases. **B**, Enlarged view of the human atria with different anatomical regions indicated by different colors. **C**, Relative sodium conductance (G_Na_). **D**, Relative gap junctional coupling efficiency (G_gap_). **E**, Unipolar electrogram from the bulk atrial tissue showing alternating slow and fast pacing frequencies corresponding to sinus rhythm (SR) and reentrant driver frequencies. **F-G**, Unipolar electrograms next to the circuit of trapped reentry showing fractionation during SR.


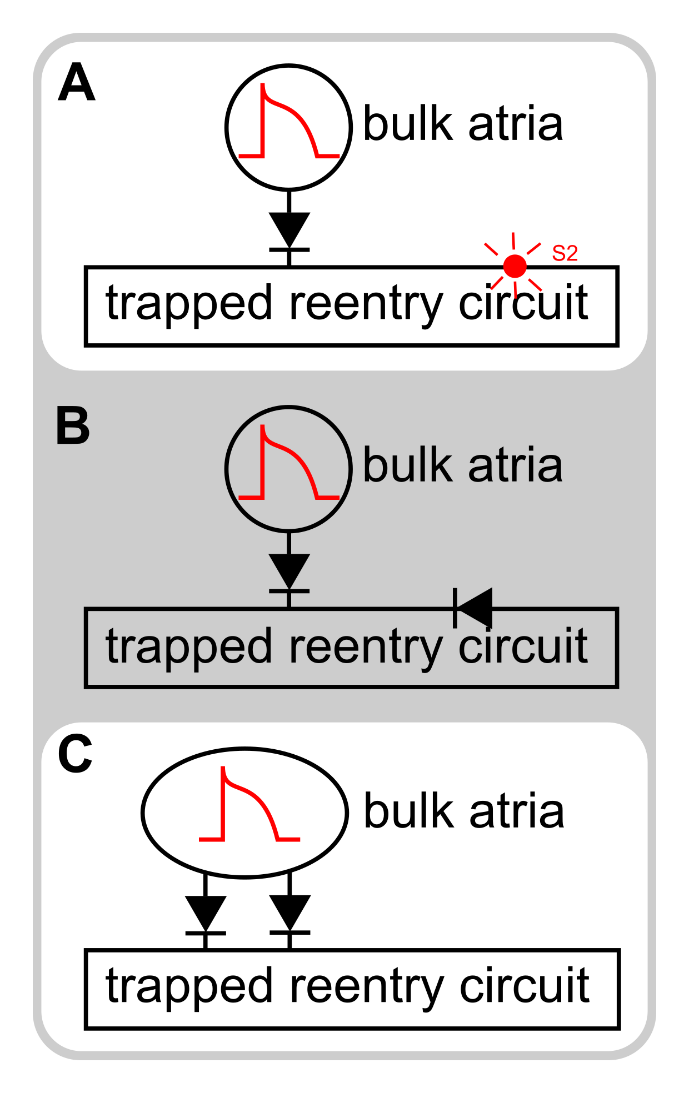
**Figure S7. Alternative schematic circuit designs to create trapped reentry.** An analogy was made with an electrical circuit, where the sinoatrial node and bulk atrial tissue are depicted by a circle with a simplified action potential, and the entrance to the circuit is denoted by a diode, signifying unidirectional block **A**, The design in the main manuscript, which uses an S2 pulse to create reentry. **B**, A design making use of two unidirectional blocks to create reentry. **C**, A design making use of two unidirectional entrances that can start reentry.

**ONLINE TABLES AND SUPPORTING INFORMATION**

**Table T1. Parameters for circuits of trapped reentry in all three human atrial models.** Parameters are provided for excitation waves to enter (Entrance), get trapped in (Confinement) and escape from (Release) the long and short circuits of trapped reentry shown in Figure S4. CRN: Courtemanche model, CRN AF: Courtemanche AF model, G_Na_: sodium conductance. D: diffusion tensor.

| Circuit | Long | | | | Short | | |
| --- | --- | --- | --- | --- | --- | --- | --- |
| Model | CRN | CRN AF | Loewe | CRN | | CRN AF | Loewe |
|  | Entrance | | | | | | |
| G_Na_-min (%) | 31.4 | 32.1 | 34.8 | 31.4 | | 32.1 | 34.8 |
| G_Na_-max (%) | 100.0 | 100.0 | 100.0 | 100.0 | | 100.0 | 100.0 |
|  | Confinement | | | | | | |
| G_Na_-S2 (%) | 80.0 | 100.0 | 100.0 | N/A | | 80.0 | 100.0 |
| G_Na_-min (%) | 30.6 | 29.5 | 32.5 | N/A | | 37.4 | 32.7 |
| G_Na_-max (%) | 41.3 | 42.3 | 45.5 | N/A | | 42.4 | 45.5 |
|  | Release | | | | | | |
| G_Na_-min (%) | 37.0 | 39.0 | 43.0 | N/A | | 39.0 | 44.0 |
| G_Na_-max (%) | 41.3 | 42.3 | 45.5 | N/A | | 42.4 | 45.5 |
| D-min (%) | 20.0 | 10.0 | 30.0 | N/A | | 10.0 | 30.0 |
| D-max (%) | 90.0 | 90.0 | 90.0 | N/A | | 90.0 | 90.0 |

**SUPPLEMENTARY VIDEO LEGENDS**

**Video V1: 2D *in silico* trapping and release of excitation waves**: Two-dimensional *in silico* optogenetic model of trapped reentry (circuit with d_2_/d_1_ = 2.45 from Figure 3) in virtual NRAM cultures with different G_gap_ (top row) and G_Na_ (bottom row). Blocking (0% release), full release on every rotation (100% release) and intermediate regimes (e.g. 25% release) can be seen. See Figure 1 for additional details.

**Video V2: Experimental trapping of excitation waves in an NRAM culture**: Essential steps to experimentally realize trapped reentry (Experiments shown in Figure 2A-2F and 2I). Voltage signals are made visible by means of a fluorescent dye. The timer indicates real-time elapsed seconds. The drawings at the bottom depict the sequence of different illumination patterns used to generate the camera output shown at the top.

**Video V3: Sinus rhythm under experimental trapping of excitation waves in an NRAM culture:** Long version of the experimental realization of trapped reentry. Voltage signals are made visible by means of a fluorescent dye. Sinus pulses are indicated by text. The timer indicates real-time elapsed seconds. The drawings at the bottom depict the sequence of different illumination patterns used to generate the camera output shown at the top.

**Video V4: Sinus rhythm through a narrow funnel in an NRAM culture:**

Wave propagation through a funnel (0.07 cm) with a straight border (Experiments shown in Figure 2G, 2H and 2I). Voltage signals are made visible by means of a fluorescent dye. Sinus pulses are indicated by text. The timer indicates real-time elapsed seconds.

**Video V5: 2D *in silico* circuit skipping of excitation waves under sinus rhythm:**

Sinus pacing at 10 Hz prevents waves from entering the circuit of trapped reentry circuit at 60% G_Na_. The upper video corresponds to an optogenetically created circuit with d_2_/d_1_ = 3.11 from Figure 3, while the lower video represents a circuit created by fibrosis with d_2_/d_1_ = 39.7 from Figure 3. The timer indicates real-time elapsed seconds.

**Video V6: 2D *in silico* sinus rhythm cannot enter a circuit of trapped reentry:**

Initiation of trapped reentry with an S1 pulse in the bulk tissue for circuits realized via optogenetic and fibrotic conduction blocks. The upper videos correspond to an optogenetically created circuit with d_2_/d_1_ = 3.11 from Figure 3, while the lower videos represent a circuit created by fibrosis with d_2_/d_1_ = 39.7 from Figure 3. After letting the reentry settle for 2 seconds, sinus pulses were given at various frequencies for 5 seconds. The timer indicates real-time elapsed seconds.

**Video V7: 3D funnel design**: Three-dimensional rotational view of the funnel that was used to connect the circuit of trapped reentry to the bulk of the atria in the realistic whole atria simulations of Figure 4. The orientation of the first frame is such that you are watching from the inside of the circuit towards the outside through the funnel opening. The epicardial and endocardial surfaces are located above and below the funnel, respectively.

**Video V8: 3D trapping of excitation waves**: Three-dimensional trapping and release of an excitation wave in the whole human atria corresponding to the process shown in Figure 6. To show the geometry of the entire atria, a 360 degrees rotation is included. The movie has been slowed down 10 times (the timer indicates real-time seconds) to clearly see trapping and release of the excitation waves.

**Video V9: Virtual unipolar electrograms indicating the presence of trapped reentry in 3D**: Simultaneous visualization (the timer indicates real-time seconds) of trapped reentry voltage maps and the accompanying unipolar electrograms at three of the locations shown in Figure 7. Please note that the recorded signals appear in the same time (12 seconds) as they would in a human heart containing a circuit of trapped reentry.

**SUPPLEMENTARY METHODS**

1. **Experimental methods**

**1.1) Preparation of CatCH-expressing monolayers:** Monolayers of neonatal rat atrial cardiomyocytes (NRAMs) expressing Ca^2+^-translocating channelrhodopsin (CatCh) were generated as follows. Hearts were excised from anaesthetized 2-day-old Wistar rats (RRID:RGD_737929). The atria were cut into small pieces and dissociated in a solution containing 450 U/ml collagenase type I (Worthington, Lakewood, NJ) and 18.75 Kunitz/ml DNase I (Sigma-Aldrich, St. Louis, MO). The resulting cell suspension was enriched for cardiomyocytes by preplating for 120 minutes in a humidified incubator at 37°C and 5% CO_2_ using Primaria culture dishes (Becton Dickinson, Breda, the Netherlands). These cells were seeded on round glass coverslips (15-mm diameter; Gerhard Menzel, Braunschweig, Germany) coated with bovine fibronectin (100 μg/ml; Sigma-Aldrich) to establish monolayers as previously described^18^. After incubation overnight in an atmosphere of humidified 95% air - 5% CO_2_ at 37°C, these monolayers were treated with Mitomycin-C (10 μg/ml; Sigma-Aldrich) for 2 hours to minimize proliferation of the remaining non-cardiomyocytes. At day 4 of culture, the NRAM monolayers were incubated for 20-24 hours with CatCh-encoding lentiviral vector particles at a dose resulting in transduction of nearly 100% of the cells. Next, the cultures were washed once with phosphate-buffer saline, given fresh culture medium and kept under culture conditions for 3-4 additional days.

**1.2) Optical mapping and optogenetic manipulation:** Optical voltage mapping was used to investigate trapping and releasing of excitation waves in the CatCh-expressing monolayers on day 7 of culture by using the voltage-sensitive dye di-4-ANBDQBS (52.5 μM final concentration; ITK diagnostics, Uithoorn, the Netherlands). Optical data were acquired using a MiCAM ULTIMA-L imaging system (SciMedia, CostaMesa, CA) and analyzed with BrainVision Analyzer 1101 software (Brainvision, Tokyo, Japan). Only monolayers showing homogeneous transgene expression and uniform action potential (AP) propagation at 1-Hz pacing were included for the optogenetic investigation (n=8). CatCh was locally activated by using a patterned digital mirror device (Polygon400; Mightex Systems, Toronto, ON) connected to a 470-nm, high-power collimator light-emitting diode (LED) source (50 W, type-H, also from Mightex Systems). PolyLite software (Mightex Systems) was used to control the location of the areas of illumination.

**1.3) *In vitro* realization of trapped reentry:** The set-up for the experimental initiation process of trapped reentry is shown in Figure S1. The movie frames at the bottom depict the patterns that were uploaded into the patterned illuminator and resulted in the representative optical mapping data presented in Figure 2A and 2B.

1. Pattern 1 shows the closed reentry circuit, which upon realization through patterned illumination, initially creates outward waves that move away from the illuminated area. Once these waves have passed, an effective non-conducting area is established in the illuminated parts of the NRAM cultures.
2. Pattern 2 shows the S1 pulse consisting of a small “bridge” between the outer illuminated ring and the inner illuminated core. The S1 pulse initiates excitation waves in both directions of the circuit, which will bump into and annihilate each other at the opposite side in the circuit. The depolarizing photocurrent delivered in the area of the bridge will render this part of the monolayer non-conducting.
3. Pattern 3 is similar to pattern 2 except for a slight leftward extension of the bridge. Pattern 3 is imposed when the refractory period after the S1 pulse has ended. As a result, a single wave will appear that runs counterclockwise, but would get blocked in the clockwise direction due to the presence of the bridge.
4. Pattern 4 shows the removal of the bridge before arrival of the excitation wave front. This allows the wave that was created with pattern 3 to freely go around the inner illuminated core and become reentrant. Sinus rhythm is maintained in the area outside of the outer illuminated ring by 1-Hz pacing at the indicated spot.
5. Pattern 5 is a continuation of pattern 4, where there is a reentry present in the inner circuit.
6. Pattern 6 shows a small funnel in the outer illuminated ring. This funnel creates an area where conduction is possible again. However, the funnel proved too narrow to allow escape of the reentrant wave.
7. Pattern 7 has a slightly wider funnel than pattern 6.As a consequence, the conduction area is wide enough to overcome the source-sink mismatch and to frequently let the reentrant wave escape.
8. Pattern 8 shows an even wider funnel in the outer illuminated ring, causing release of the reentrant wave on every rotation around the inner illuminated core.
9. **Computational methods**

**2.1) Simulation set-up:** The numerical solver was implemented with the C and C++ programming languages, using the CUDA toolkit for performing the majority of computations on graphics processing units (GPUs). Visualization of results was done with the help of the Python programming language and ParaView (Kitware). Computations were performed with single precision and run on an AMD Ryzen Threadripper 2950X 16-Core machine with two GeForce RTX 2080 Ti graphics cards.

For the simulations of the whole atria with a spatial resolution of 150 and 100 μm, one GPU did not suffice to store all the variables needed in the computation. By making use of unified memory, available on the newest NVIDIA architectures, it was possible to run simulations on two coupled GPUs.

**2.2) Cell models:** Different cell models were used for two-dimensional (2D) and three-dimensional (3D) simulations of the trapped reentry phenomenon as described below.

The 10 major ionic currents that make up I_ion_ in the 2D model of Majumder *et al.*^22^ (Eq. 1 of the main manuscript) are:

|  | $I_{ion}=I_{Na}+I_{CaL}+I_{K1}+I_{to}+I_{Ksus}+I_{Kb}+I_{Nab}+I_{Cab}+I_{f}+I_{KACh}$ | (Eq.S1) |
| --- | --- | --- |

Here, the different cardiac currents are represented as follows: the fast Na^+^ current (I_Na_), the L-type Ca^2+^ current (I_CaL_), the inward rectifier K^+^ current (I_K1_), the transient outward K^+^ current (I_to_), the sustained outward K^+^ current (I_Ksus_), the background K^+^ (I_Kb_), the background Na^+^ (I_Nab_) and the background Ca^2+^ (I_Cab_) currents, the hyperpolarization-activated funny current (I_f_), and the acetylcholine-mediated K^+^ current (I_KACh_). Units for conductance measurements (G_X_) and measurements of intracellular and extracellular ionic concentrations ([X]_i_ and [X]_o_), are in nanosiemens per picofarad (nS/pF) and millimole per liter (mM), respectively.

In the three 3D human atrial models developed by Courtemanche *et al.*^25,26^ and Loewe *et al.*^27^, the total ionic current is given by:

|  | $I_{ion}=I_{Na}+I_{CaL}+I_{K1}+I_{to}+I_{NaCa}+I_{NaK}+I_{Kr}+I_{Ks}+I_{bNa}+I_{bCa}+I_{pCa}+I_{Kur}$ | (Eq.S2) |
| --- | --- | --- |

Here, the different cardiac currents are represented as follows: the fast Na^+^ current (I_Na_), the L-type Ca^2+^ current (I_CaL_), the inward-rectifier K^+^ current (I_K1_), the transient outward K^+^ current (I_to_), the Na^+^/Ca^2+^ exchanger current (I_NaCa_), the Na^+^/K^+^ pump current (I_NaK_), the rapid-delayed rectifier K+ current (I_Kr_), the slow-delayed rectifier K^+^ current (I_Ks_), the background Na^+^ current (I_bNa_), the background Ca^2+^ current (I_bCa_), the plateau Ca^2+^ current (I_pCa_), and the ultra-rapid-delayed rectifier K^+^ current (I_Kur_). Units for conductance measurements (G_X_) and measurements of intracellular and extracellular ionic concentrations ([X]_i_ and [X]_o_), are again in nS/pF and mM, respectively.

**2.3) Simulation technical details:** Simulations were performed both in 2D and 3D.

*Monolayer*: In the 2D NRAM model, isotropy was assumed, and thus the diffusion tensor (a measure of gap junctional coupling efficiency) takes on a diagonal form with identical elements. Therefore **D** reduced to a scalar in our calculations, with a value of 0.00012 cm^2^/ms. This resulted in a signal conduction velocity of 19 cm/s. It has been shown before that anisotropy in monolayers amounts to a rescaling of the results in a homogenous monolayer^49^, which is why simulations were performed in homogeneous tissue.

Eq.1 was integrated in time using the forward Euler method with time step Δt = 0.005 ms, and in space, using the centered finite-differencing scheme with space step Δx = Δy = 0.003125 cm, subject to “no flux” boundary conditions. The simulation domains contained a circular region with a diameter of 486 pixels cut from a square containing 512 × 512 grid points. By doing so, the simulated tissue had approximately the same diameter as a well of a 24-well cell culture plate. The gating variables in the electrophysiological model for the NRAMs were integrated using the Rush and Larsen scheme^50^.

*Whole organ*: Owing to the natural anisotropy of realistic cardiac tissue, the elements were computed on the basis of a reconstructed fiber direction field. The transverse diffusion coefficient (D_t_, for signal propagation across the fibers) was assumed to be 9 times less than the longitudinal diffusion coefficient (D_l_, for signal propagation along the fibers). Elements of the diffusion tensor were computed as follows:

|  | $D_{ij}=\left( D_{l}-D_{t} \right)\alpha_{i}\alpha_{j}+D_{t}\delta_{ij}$ | (Eq.S3) |
| --- | --- | --- |

where α_i_ are components of the unit vector that is oriented along the direction of a fiber. We used D_l_ = 1.54 cm^2^/s. This resulted in a conduction velocity of 72 cm/s along the fiber direction.

Eq.1 was integrated in time using the forward Euler method with time step Δt = 0.005 ms, and in space, using the centered finite-differencing scheme with space steps Δx = Δy = 0.0150 and 0.0100 cm in whole atria, subject to “no flux” boundary conditions. The simulation domains for the whole atria contained 17.391.128 and 58.695.057 grid points for a resolution of 150 and 100 μm, respectively. The gating variables in the electrophysiological model for the human cardiomyocyte were integrated using the Rush and Larsen scheme^50^.

**2.4) APs of human atrial myocyte models:** The 3D atrial simulations were carried out with three different models (Figure S2), each of them describing a different stage in atrial remodeling. When simulating with realistic cellular models, they have to be pre-paced such that they settle into an AP duration (APD) that doesn't change from beat to beat. This was done by delivering 50 pulses at a frequency of 1 Hz at the single-cell level, after which the recorded parameters were used to initialize all cells in the 3D models.

Figure S2 shows the APs of all three models after 50 pulses at the single-cell level. A healthy atrial cell has a notch-dome AP and is described by the Courtemanche model^25^. The electrical remodeling in early stages of atrial fibrillation (AF) is characterized by APD shortening and AP triangulation with slow repolarization as modelled in the Courtemanche AF model^26^. Under chronic AF remodeling as described by Loewe^27^, the AP stays initially at a higher potential, but has fast repolarization, making its APD_90_ shorter than the AF remodeled AP. These differences in AP result in smaller reentry circuits supporting remodeled APs.

**2.5) Electrogram computation:** Because of the monodomain formulation of the cell models, I_m_ from Eq. 2 in the main manuscript is readily available and allows us to compute the extracellular potential ϕ_e_. Since ϕ_e_ is linearly related to V_m_, it is computed for practical reasons as a weighted sum of the membrane potential V_m_ using the discretized diffusion operator, which was already defined to solve the propagation equation (Eq.1 in the main manuscript).

1. **Electrically isolated circuit design methods:**

**3.1) 2D funnel design:** The funnel connecting the inner region of the trapped reentry circuit to the bulk tissue can be characterized by means of two lengths (Figure 3A). These are the arclengths of the funnel along the inner and the outer radius of the funnel, denoting the surfaces where the funnel touches the inner circuit and the bulk of the tissue, respectively. The ratio between these two lengths is a measure of the source-sink mismatch present in a particular funnel and affects the ability to excite the tissue at the inside and outside openings of the funnel. The funnel that was used for the simulations in Figure 1 had a d_2_/d_1_ ratio of 2.45.

By making d_2_ larger than d_1_, a somewhat triangular shape is created, whose base is on the outside. Going from outside the circuit to inside, the number of cells that needs to be excited decreases, which ensures that there will always be enough source to supply the sink and hence allow wave propagation. However, going from inside the circuit to the outside, the number of cells that needs to be excited increases. In this situation, there is more sink than source, which prevents wave propagation if the source-sink mismatch is large enough.

**3.2) 3D funnel design:** The design of the funnel that was used in all 3D simulations is shown in Figure 4A and Supplementary Video V7. The leftmost panel of Figure 4A shows an image of the whole human atria. Zooming in on a specific part of the right atrium, the shorter circuit that was used for trapped reentry becomes clearly visible. On the left side of the circuit, the funnel can be seen, which is of crucial importance to accomplish the effect of trapped reentry.

The third panel zooms in on the top view of the funnel. For visualization purposes, only the contour of the funnel has been plotted. The bulk of atrial tissue and the reentry circuit are situated on the left and right side of the funnel, respectively. Going from outside to inside the circuit, a gradual increase in funnel width was designed. This particular design allows excitation waves to enter the circuit even when G_Na_ drops. Going from inside the circuit to outside, the funnel width gradually decreases up to the point where the funnel connects to the bulk of atrial tissue. Due to the resulting source-sink mismatch, this sudden opening towards a bulk of cardiomyocytes causes conduction block. To increase the number of myocytes that becomes available for the excitation wave, the exit of the funnel was designed to have a sharp angle with the funnel wall, i.e. to bend back towards the fibrotic region. Collectively, these features create the conditions for unidirectional conduction block following a drop in G_Na_.

The side view of the funnel can be seen in the rightmost panel of Figure 4A. The epicardial surface and atrial lumen are located above and below the funnel, respectively. Going from outside to inside the circuit, one can see a smaller increase in the funnel height than in the funnel width due to the small atrial wall thickness. When going from inside the circuit to outside, a gradual decrease in funnel height is followed by a sudden increase in funnel height caused by a vertical cut-off, i.e. a right angle with the fibrotic region.

To determine the radius needed for obtaining conduction block in two- and three-dimensional funnels, we based ourselves on Thomas *et al*.^44^ The smallest width of the funnel (Figure 4A, top view) is 7 voxels, which, at the spatial resolution of 150 μm, corresponds to 1.05 mm. The smallest height of the funnel (see Figure 4A, side view) is 8 voxels, which amounts to 1.20 mm. The minimal cross-sectional area of the funnel is therefore 1.26 mm^2^. While this is rather large and *in vivo* scenarios will include smaller minimal cross-sectional areas, this larger area was chosen on purpose to exclude any possible discretization effects. The larger funnel opening permitted us to ascribe the unidirectional conduction block solely to reductions in G_Na_ and G_gap_.

The values that were used for the funnel opening are also dependent on the fiber orientation inside the atria. Therefore, depending on the orientation of the circuit in the atria, the funnel opening will need to be slightly modified. In the second and third panel of Figure 4A, the fibers are oriented from the upper left corner towards the lower right corner.

**3.3) Trapped reentry circuit in full human atria:** To fully appreciate the geometry of the whole human atrium, a rotational view has been provided (Figure 4B) with different anatomical regions shown in different colors. In this way, the crista terminalis, Bachmann's bundle, pulmonary veins, pectinate muscles as well as the right and left atrial appendages can be easily distinguished. In both panels, a trapped reentry circuit (indicated by arrows) was included in the right atrial wall. Fibrotic tissue was used to create non-conducting regions in these circuits. The human atria in the model have dimensions of 10.755 cm × 8.895 cm × 7.035 cm. The size of the circuit (inner obstacle plus conducting region) is 2.694 cm × 1.8375 cm × local wall thickness (i.e. ~4.95 cm^2^ × local wall thickness), and of the inner obstacle is 1.410 cm × 0.468 cm × local wall thickness (i.e. ~0.66 cm^2^ × local wall thickness).

Figure 4B1 shows the atria in their conventional view. In the center of the circle, a frontal view of the atria is shown, in which the right atrium (left side of the figure), left atrium (right side of the figure) and pulmonary veins (orange pink regions) are clearly visible. Around it, a series of rotated views (36 degrees each) is depicted. Unfortunately, in this view the trapped reentry circuit is barely visible.

Therefore, another view of the atria has been provided in Figure 4B2 for optimal visualization of the trapped reentry circuit. Also here, a series of rotated views (36 degrees each) is shown.

1. **Trapped Reentry parameters motivation and methods:**

**4.1) Enabling or disabling wave escape based on source-sink mismatch:** To motivate our choice for the design of the entrance to the trapped reentry circuit, as well as the changes in global parameters that were investigated in the manuscript, it is useful to take a look at the key concept of the source-sink relationship related to the propagating activation wavefront^51^. A wavefront can propagate as long as unexcited but excitable cells (the "sink") have their *sodium channels* activated by the diffusion current moving forward from depolarized cells at the leading edge of the front (the "source"). At the same time, the *gap junctional coupling* (mediated through the diffusion current) acts as a drain on the source. If a relatively small source is attached to a larger sink, the loss of source current caused by the sink may reduce the current available for excitation to the point that propagation fails. This indicates a critical relationship between the source current for excitation and the mass of tissue being excited, which drains the source current electrotonically. This principle can be used to create an isthmus capable of unidirectional block under the right circumstances. This can be accomplished in two ways through 1) ionic changes or 2) geometrical changes.

Let’s first take a closer look at the ionic changes that can create unidirectional block. By decreasing the sodium channel conductance (G_Na_⬊), excitation of the sink gets reduced which results in blocking of the wave at the funnel opening. On the other hand, if we reduce the gap junctional coupling (G_gap_⬊), there is a longer exposure of the cells in front of the wavefront to the diffusion current such that the source doesn't get immediately drained, making propagation possible again.

Another possibility lies in changing the geometry of the opening. If the d_2_/d_1_ ratio of the funnel gets increased (Figure 3), more sink is created for the outgoing wave. When this ratio becomes large enough, conduction block will happen because there is not sufficient source to supply the sink. This process can be reversed by making the ratio smaller again.

The described processes give rise to realistic situations^35^ that can create trapped reentry *in vivo*. The first one is a decrease in G_Na_ combined with an ectopic pulse to initiate trapped reentry^31,32,33,52,53^, and a reduction in G_gap_ to release^34,54^ the trapped waves (Figure 1, 5, 6, S6). The second one would be one where the fibrotic pattern grows in such a way that it creates a funnel as an opening with unidirectional block. In combination with an ectopic pulse, this gives once again rise to trapped reentry. The fibrotic pattern can, however, grow in such a way that the opening of the funnel on the outside becomes smaller, opening the way for escape of the trapped waves (Figure 2 and 3). Combinations of these two proposed scenarios are possible as well.

**4.2) 2D Trapped reentry technique comparison:**

In Figure 3, we demonstrated a similarity in behavior between optogenetically created trapped reentry circuits and trapped reentry circuits realized through fibrosis. Moreover, subpanels B and C of this figure show the difference between the protocols that were used to induce trapped reentry in different settings. For the experimental trapped reentry protocol (dots circled with a solid blue line), we made use of the optogenetic approach by keeping the tissue at its normal conductance levels, but changing the isthmus width (and hence the d_2_/d_1_ ratio). Going from a high to a low d_2_/d_1_ ratio, resulted in release of the reentrant waves. For the protocol that was used for the 2D simulations of Figure 1, we applied optogenetics *in silico* and geometry d5 with a d_2_/d_1_ ratio of 2.45 from Figure 3. Excitation wave trapping occurred when G_Na_ was lowered. For the 3D simulations, we used the fibrotic approach. Also here, trapping occurred by lowering the G_Na_ level.

**4.3) 2D Trapped reentry influence of sinus wave frequency:**

Entering a trapped reentry circuit gives a small deflection in the electrogram when measured right on top. To investigate whether a trapped reentry circuit could be detected early on through sinus pulses alone, the virtual NRAM tissue was paced at different frequencies under optogenetic and fibrotic excitation block conditions (Figure S3). We observed that at certain frequencies, the circuit got skipped altogether rendering it essentially “invisible”. The location of sinus pacing in a well, together with the two possible outcomes of entering or skipping the circuit, are shown in Figure S3A. The entering percentage decreased with increasing pacing frequencies (Figure S3B), meaning that the reentrant circuit gets skipped at higher frequencies. A complete analysis of 4 selected frequencies (2, 5, 10 and 12.5 Hz) for each of the 15 funnel geometries and for different G_Na_, is shown in Figure S3C. The results of this experiment imply that trapped reentry circuits might get missed altogether when looking for them.

**4.4) 3D Trapped reentry parameter details:** Trapped reentry was realized in two different reentrant circuits in the human atria (Figure S4). The funnel in these circuits was the same (Figure 4), but the inner circuit was different such that the travel time of the waves would differ for one revolution through the circuit. The shorter circuit supported reentry at higher frequencies (4.7 Hz) than the longer one (2.9 Hz).

For both circuits, all three human atrial models were tested, see section 2.4 above. The images that are shown on the left are both visualizations of the release phase in the Courtemanche AF model.

The graphs in Figure S4 give details about the datapoints used in Figure 5. In each graph the top row of points shows the largest value of G_Na_ for which it was possible to trap the excitation wave at a diffusion coefficient of 100%, i.e. a normal level of gap junctional coupling. Stepwise reduction of the diffusion coefficient leads to progressively more release of the trapped excitation waves until gap junctional coupling efficiency is so low that it interferes with normal wave propagation. Measurement points were plotted at 10 and 1% resolution for G_gap_ and G_Na_, respectively. The upper boundary of G_Na_ was also included, which was measured at a sensitivity of 0.1%.

To construct Figure 5, we looked at the regions showing escape of the trapped wave to deduce the range of diffusion coefficients for which release was possible. This was done because enlarging the circuit turned 50% release into 100% release.

More details about the parameters for trapped reentry can be found in Table T1. It contains all the values that resulted from scanning for all stages of trapped reentry, i.e. entrance of the circuit, trapping of the excitation wave inside the circuit and release of the excitation wave from the circuit. In Table T1, G_Na_-S2 is the sodium conductance level needed to allow a wave to be trapped inside the circuit after the ectopic S2 pulse that was given to initiate reentry. The values in the four bottom rows of Table T1 are derived from Figure S4.

**4.5) 3D Trapped reentry spatial resolution:** In Figure S4, no mention was made about the lower boundary of the parameter range. This is because the lower range is mainly determined by the spatial resolution at which simulations are carried out. To show this, parameter ranges were calculated for two different spatial resolutions: 150 and 100 μm. This was done for the shorter circuit shown in Figure S4 using the Courtemanche AF model.

The results are visualized in Figure S5. The top row shows the trapped reentry circuit in a 3D slice of the right atrium. The size difference between the left and right atrial slice is determined by how many points are inside the simulation domain. For a small space-step (i.e. a high spatial resolution), more voxels will be needed to simulate the full circuit. To carry out these large simulations, we resorted to computing on two GPUs simultaneously. The use of different spatial resolutions revealed larger parameter regions supporting trapped reentry at the higher spatial resolution.

**4.6) 3D Unipolar electrograms overview:** Unipolar electrograms were not only taken at the three points depicted in Figures 6 and S6 in the main manuscript. In total 85 electrograms were taken at the epicardial surface of the atria. They were placed in selected areas. For the stimulation protocol that was used for Figure 6, 10 representative electrograms are shown in Figure 7. These selected electrograms come from six different areas, which are color coded in the figure (red, dark green, orange, light green, black). Each color corresponds to a different location in- or outside the trapped reentry circuit. Each location displays particular characteristics as can be seen in the corresponding electrograms:

1. *red*: Inner fibrotic region of the reentry circuit. Here, no direct propagation of an electrical signal is witnessed. However, the far field effects of the circuit and of the sinus pulses are visible on the electrograms and display fractionated behavior.
2. *dark green*: Conducting tissue inside the reentry circuit. In these electrograms, it is possible to see the high frequency of wave propagation once trapped reentry has commenced.
3. *orange*: Outer fibrotic region of the reentry circuit. Just like for the inner fibrotic region, the electrograms show far field effects. Fractionation due to trapped reentry can once again be observed.
4. *light green*: Region just outside the reentry circuit. Although the measurement points are extremely close to the reentry circuit, the resulting electrograms show hardly any signs of trapped reentry, but predominantly SR. These electrograms illustrate that the effect of trapped reentry can easily go unnoticed.
5. *black*: Region far away from the reentry circuit, including measurement points in the left atrium. Here, the electrograms show absolutely no sign of the presence of the trapped reentry circuit. This showcases that a dormant arrhythmia might be present in the atria while the majority of electrogram signals displays SR.

Please note that the amplitude of the “red” and “orange” signals is lower than those obtained from the other regions. This is a direct result of the electrode not connecting to conducting tissue and therefore only picking up far field effects. All other regions show high spikes whenever the propagation wave passes.

**4.7) Alternative methods to create a trapped reentry circuit:** To explain the principle of trapped reentry, it was mentioned that one ectopic beat was needed to create a reentrant circuit to start trapped reentry. However, it has been shown that other mechanisms might be able to initiate this process as well, such as the occurrence of two ectopic foci within the circuit^55^. Other possibilities exist as well (Figure S7). As an equivalent symbol for the sinoatrial node (SAN) which periodically sends propagation waves into the bulk atria, a circle was taken with in it a simplified action potential. Funnels capable of unidirectional block can be depicted as diodes (triangle with line). In an electrical circuit, the block imposed by a diode can be overcome by providing a large enough voltage, similar to how we are able to influence source-sink mismatch.

With these pictorial representations, it is now easy to draw schemes of other possibilities to create trapped reentry. One of these possibilities is to have two regions of unidirectional block (Figure S7B). One would be at the entrance of the circuit and another one would be within the circuit. In this way, it is possible for the reentry to establish from a wave originating from the SAN without the need of an S2 interference. Another possibility entails multiple entrances all having the property of unidirectional block. By means of the interplay of arrival times to the different entrances, this might also constitute an alternative way of initiating the trapped wave. More possibilities can be envisioned indicating that the chances for observing this phenomenon are larger than what is shown here.

**SUPPLEMENTARY REFERENCES**

1. Pertsov AM, Davidenko JM, Salomonsz R, Baxter WT, Jalife J. Spiral waves of excitation underlie reentrant activity in isolated cardiac muscle. *Circ Res.* 1993;72(3):631-50
2. Rush S, Larsen H. A practical algorithm for solving dynamic membrane equations. *IEEE Trans on Biomed Eng*. 1978;BME-25(4).
3. Comtois P, Kneller J, Nattel S. Of circles and spirals: bridging the gap between the leading circle and spiral wave concepts of cardiac reentry. *Europace*. 2005;7(s2):S10–S20.
4. King JH, Huang CLH, Fraser JA. Determinants of myocardial conduction velocity: implications for arrhythmogenesis. *Front Physiol*. 2013;4:154.
5. Klabunde RE. Cardiac electrophysiology: normal and ischemic ionic currents and the ecg. *Adv Physiol Educ*. 2017;41(1):29–37.
6. Spach MS, Dolber PC, Heidlage JF. Influence of the passive anisotropic properties on directional differences in propagation following modification of the sodium conductance in human atrial muscle. a model of reentry based on anisotropic discontinuous propagation. *Circ Res*. 1988;62(4):811–832.
7. Nagai Y, González H, Shrier A, Glass L. Paroxysmal starting and stopping of circulating waves in excitable media. *Phys Rev Lett*. 2000;84(18):4248.
